# The Feedback Loop Between the Demand for Voluntary Private Insurance and the Burden of Healthcare System: An Explanatory System Dynamics Model of Hong Kong

**DOI:** 10.34172/ijhpm.2022.6738

**Published:** 2022-07-06

**Authors:** Yiran Zou, Junqiao Chen

**Affiliations:** ^1^Center for Data Science, New York University, New York City, NY, USA.; ^2^Roche Hong Kong and Macau, Hong Kong SAR, China.; ^3^University Institute of Lisbon (ISCTE-IUL), Lisbon, Portugal.; ^4^Faculty of Sciences, University of Lisbon, Lisbon, Portugal.

**Keywords:** Private Health Insurance, Universal Healthcare, Waiting Time, Feedback Loop, System Dynamics, China

## Abstract

**Background:** Many countries with universal healthcare have a parallel private healthcare sector due to the waiting time in the public sector. People purchase individual health insurance to pay for private services. Past studies on the relationship between the public sector’s waiting time and the demand for health insurance have two limitations: not considering the capacity of the private sector, and subsequently, the omission of a feedback loop. These limitations are also present in the health insurance policy discussion in Hong Kong, where the public sector is overstretched. A lack of understanding of market dynamics might lead to unrealistic expectations of public policy. This study highlights these limitations, and tries to answer the research question: whether the historical dynamics between the intersectoral imbalance of burden and the demand for health insurance in Hong Kong could be quantitatively explained.

**Methods:** A system dynamics model was created based on a negative feedback loop. The model’s initial input was the percentage of population with health insurance in 2009, and to simulate the percentage continuously until 2019. Results from 2015 to 2019 were compared with actual figures to examine the model’s explanatory power. Multivariable sensitivity analysis was performed.

**Results:** With initial fluctuation, the simulated result stabilized and was within the acceptable error range from 2015 to 2019. The mean absolute percentage error (MAPE) was 0.94%. At the end of 2019, the simulated percentage of population with health insurance is 36.6% versus the "real value" of 36.7%. Simulated patient admissions and occupancy rates also approximate the reality. Sensitivity analysis demonstrates the robustness of the model.

**Conclusion:** We can quantitatively explain the feedback loop between health system burden and demand for health insurance. With local parameterization, this model should be transferable to other universal health systems for a better understanding of the system dynamics and more informed policy-making.

## Background

###  Demand for Voluntary Private Health Insurance in a Universal Healthcare System

**Key Messages**

** Implications for policy makers**

- In making policy to shift some of the burden from the public healthcare sector to the private sector, the government must consider the capacity of the private sector and the subsequent feedback loop in such a market dynamic.
- Specifically, in making policy to promote the purchase of voluntary private health insurance, the government must consider the feedback loop between intersectoral imbalance and the demand for health insurance. This feedback loop has been quantitatively explained in this model.
- Because of a negative feedback loop, there is an upper limit on the percentage of population with private health insurance: more people with private insurance reduces the difference in waiting time between the public and private sectors, therefore reducing the attractiveness of private insurance.
- A tax incentive policy might boost the purchase of private health insurance initially, but its effect will be moderated by the negative feedback loop. Other accompanying policies are needed to reduce the burden on the public healthcare sector and benefit the entire population.
- In a universal health system, the *current* percentage of population with private health insurance is often not readily available due to its commercial and voluntary nature. The government may adopt this model to estimate the *current* percentage if it has one historical value of the percentage five to ten years ago, and the healthcare resource and utilization data from then to now. This model can account for the effect of a tax incentive policy, and the model users only need to quantify the policy as average tax saving per capita.

** Implications for the public**

This study might benefit the public by better policy-making from the government. Using data from Hong Kong, we explained the market dynamics of waiting time in the public sector, waiting time in the private sector, and the demand for private health insurance. We explained a negative feedback loop in which more people with private insurance reduce the difference in waiting time between the public and private sectors, which then reduces the attractiveness of private insurance. The effect of a policy tax incentive on purchasing private insurance is moderated by this negative feedback loop. The public might reference this study in their advocacy work with the government to examine the level of tax incentive for purchasing health insurance and, more importantly, to design accompanying policies to address the underlying issues of the overstretched public healthcare sector.

There are 73 countries that have passed legislation on universal health coverage in the world.^1^ Universal healthcare system is essentially a government-subsidized healthcare system that offers affordable healthcare services to residents. In most of these countries, the public healthcare system provides most services to the majority of the residents. If the public sector provides timely access to high-quality treatment in comfortable facilities and in a manner that reflects the preferences of patients, there will be no demand for a parallel private market. The fundamental motive for purchasing private insurance is to gain access to private providers who offer something that the public sector does not. Commonly observed worldwide, the private sector provides access to one or more of the following: better amenities while receiving medical care; greater freedom in choosing facilities or physicians; more shared decision making in treatment; and most noticeably, shorter waiting time.^2-5^

 Besides those common advantages, private providers in Hong Kong also have more choices in diagnostics and pharmaceutical treatments, while public doctors can only order those approved by the Hospital Authority.^1,6^

 Many societies with universal healthcare have a sizable portion of their population with private individual health insurance to pay for services of the private sector. Take Australia, Ireland, and Hong Kong’s figures in 2019 as examples. In Australia, 11.2 million Australians (44% of the population) had some form of private patient hospital cover, and 13.6 million (53%) had some form of general treatment coverage.^7^ In Ireland, 46% of the population has some form of health insurance.^8^ In Hong Kong, 35% of the population has individual health insurance, with most plans are designed to cover inpatient care.^9^

###  Previous Studies and Their Limitations

 Waiting time in the public sector is most noticeable among all the factors that drive the demand for voluntary private health insurance; hence it has been subject to study in several countries. The earliest one is a hallmark study published in 1999 which found a positive effect between waiting lists (as a proxy for waiting time) for treatment in the National Health Services and the purchases of private health insurance in the United Kingdom.^10^ However, this study was revisited later by another group of researchers, who found that waiting lists were not a good proxy for waiting time, and the effect of waiting time was overstated.^11^ These researchers went on to study the effect of waiting time for elective surgery on private health insurance in Australia. They found that while the average expected waiting time does not increase the probability of buying insurance, a high probability of experiencing a long wait does. Another study conducted on elderly veterans of the United States found that outpatient waiting times in the free Veteran Health Administration increase demand for private health insurance. These studies reflect the difficulty in measuring wait time and modeling the effect.

 We observed two main limitations in previous studies based on our professional experience working in the healthcare industry, media reports from the private sector,^12^ and correspondences between Legislative Council members and officials from the executive branch^13,14^: not considering the capacity of the private sector, and subsequently, the omission of the feedback loop. The private sector operates in a market economy, and its supply of services is driven by the demand for services and constrained by available resources in the healthcare industry. They will not have many idle resources. Without considering this important aspect, previous studies only looked at waiting time in the public sector alone, assuming there was always a more attractive private sector regardless of how many people were using it. This does not reflect reality. In places like Hong Kong, where there is a general shortage of healthcare resources, waiting time in the private sector is shorter but still non-ignorable.^15^ Subsequently, previous studies did not consider the changing dynamics of the intersectoral imbalance as well as the demand for health insurance. They mostly adopted a static single-directional view and were only concerned with the impact of the public sector’s waiting time on health insurance demand at a given time, but did not consider the longitudinal bi-directional impact. In other words, the feedback loop from more health insurance penetration back to the public sector waiting time has not been noticed. Of note, this is not only a gap in research but also a gap in policy discussion in Hong Kong’s healthcare reform, which will be further elaborated below. This study aims at addressing these limitations. It is aligned with other scholars’ emphasis that attention needs to be paid to “the complexities of inter-sectoral relations and their impact on private health insurance demand.”^16^

###  Hong Kong Healthcare System and Reform

 Hong Kong, a high-income city and a Special Administration Region of China, also has a dual-track healthcare system. Being the predominant provider of secondary and tertiary healthcare services, the public sector provides around 88% of inpatient services. On the other hand, the private sector provides approximately 70% of outpatient services.^17^ Public hospitals are overstretched,^18^ causing prolonged waiting times. According to the Hong Kong Hospital Authority in 2017, waiting time for urgent and semi-urgent inpatient services ranges from 4 to 8 weeks.^18^ Waiting time for total joint replacement surgery in Hong Kong ranged from 36 to 110 months.^19^ In 2021, depending on where you lived in Hong Kong, the median waiting time for cataract surgery ranged from 10 to 23 months which is longer than any OECD (Organisation for Economic Co-operation and Development) country.^19,20^

 The public is acutely aware of the long waiting time, either through personal experience or by reading the news, as unbearable waiting time is often featured in prominent news outlets. It was reported that the long waiting time for specialist care in Hong Kong led to approximately 66% of its residents being dissatisfied with public health services, while long working hours caused doctors to quit public hospitals.^21,22^ Waiting time in the public sector continues to worsen due to the rapidly-growing aging population and lifestyle-related diseases caused by improved living standards.^17^ Shorter waiting time in the private sector has always been a marketing point for commercial insurers. It is also leveraged by the Hong Kong government in healthcare reform.

 One recent reform is the Voluntary Health Insurance Scheme (VHIS), proposed by the Food and Health Bureau of Hong Kong and launched on April 1, 2019.^23^ It links private health insurance with strong government regulation and incentives. After purchasing a Certified Plan, a VHIS holder may claim a tax deduction of up to HK $8000 (US $1027) depending on the annual premiums paid, which could help save up to HK $1200 (US $154).^24^ The consultation report commissioned by the Food and Health Bureau projected that 1 million people (13% of the population) would purchase VHIS,^17^ although it offers no convincing explanation behind the projection, nor differentiation between new subscribers and those switching from existing plans.

 Improving the design and regulation of private health insurance is one of the most important pillars of healthcare reform in Hong Kong and other universal healthcare systems. However, as reviewed above, most discussions on this reform did not consider the private sector’s capacity, which has led to complaints from a major private hospital that they cannot handle all the patients newly insured.^12^ VHIS can only boost the demand for health insurance on top of the fundamental demand, which still comes from the motives mentioned above (eg, longer waiting time in the public sector than the private sector). It is crucial to put this reform policy within the context of this core market dynamics. One key value of this paper is to explain the market dynamics. It explains the important historical context in which the reform was introduced.

###  Value of an Explanatory Model

 Explanatory models are used to investigate how a system works.^25^ From Shmueli’s classical work “To Explain or to Predict?,”^26^ explanatory modeling refers to the application of statistical models to data for testing causal hypotheses about theoretical constructs. In social sciences such as economics, statistical methods are used almost exclusively for causal explanation. The value of an explanatory model is that it can enhance our understanding of the system. Meanwhile, predictive modeling is the application of statistical or machine learning methods to data for the purpose of predicting new or future observations. There is a difference between explaining and predicting because measurable data are not accurate representations of their underlying constructs. In addition, explanatory models may have coincident indicators that are useful in explaining but not in predicting because they are simultaneous.

 This paper presents the readers with an explanatory model of the causal relationship between the inter-sectoral imbalance and the demand for health insurance. We aim at deepening readers’ understanding of this topic to enhance policy-making. The value of our model is not about precisely predicting the demand for health insurance, but revealing important market dynamics so that the public could have better-informed policy discussions in the future. This aligns with the current advocacy of high-quality evidence-based public policy research and decision making in Hong Kong,^27^ China and around the world.^28^

###  Research Question of this Paper

 The research question of this paper is whether we could quantitatively explain the historical dynamics between the intersectoral difference in burden and the demand for health insurance in Hong Kong from 2015 to 2019. An explanatory model can deepen our understanding of a system, which could facilitate better policy discussion in the future. Insights from this paper could close the gap in academic previous studies and policy discussions. The years of 2015 to 2019 represent a stable historical period before the coronavirus disease 2019 (COVID-19) pandemic. The success of answering this research question is measured by how accurate the simulated result (percentage of population with health insurance) is to the actual percentage. We aim to build a model that is simple and intuitive, so that its core concepts and structure could be applied to any universal healthcare system outside of Hong Kong.

## Methods

 Following the guideline on building an explanatory model,^26^ in this section, we first introduce the high-level theory of the demand for health insurance. Second, we present how several theoretical constructs were selected and how their relationship was hypothesized as feedback mechanisms. These feedback mechanisms are represented in a causal loop diagram. Then we explain what made us choose System Dynamics as the modeling method. Lastly, we follow reporting guidelines to describe the model and its data source transparently.

###  Guiding Theory

 The guideline on building explanatory models stresses the importance of having a theory to guide the causal explanation in an explanatory model.^26^ The guiding theory of the present model is Nyman’s theory of the demand for health insurance, a relatively new theory proposed in 2002.^29^ Conventional theory is primarily based on the expected utility theory and risk aversion, which holds that people purchase insurance because they prefer the certainty of paying a small premium to the risk of getting sick and paying a large medical bill. Conventional theory does not necessarily apply to a universal health system with a paralleled private sector because people can always “retreat” to the free option (the public sector). It also holds that any additional healthcare that consumers purchase because they have insurance is not worth the cost of producing it. In Nyman’s book, he presented a new theory that people purchase insurance to obtain additional income when they become ill. In effect, insurance companies act to transfer insurance premiums from those who remain healthy to those who become ill. This additional income generates purchases of additional high-value care, often allowing sick people to obtain life-saving care that they could not otherwise afford.

 This theory has been applied in theoretical modeling work and empirical studies in dual-track health systems such as Brazil and China.^30-32^

###  Feedback and Causal Loop Diagram

 As mentioned in the Introduction section, based on our previous experience working in the industry, media reports on private doctors and correspondences from the Legislative Council,^12,15^ there is a gap in academic literature and Hong Kong’s policy discussion. It is the omission of considering the crowdedness in the private sector. There is a limited capacity of private providers in response to the demand for services. Initially, to have a competitive edge over the public sector, the private sector will resource itself to be less crowded. At this moment, health insurance is attractive to people so they can afford to visit private providers. As the increase in usage outpaces the increase in capacity, the private sector becomes more crowded. One day if it becomes as crowded as the public sector, health insurance is not attractive anymore, and existing insurance customers might disenroll. As fewer people can afford to use the private sector, it becomes less crowded and health insurance becomes attractive again. This is a classic example of a balancing feedback loop. Feedback loops illustrate self-organization in complex systems such as healthcare.^30^

**Figure 1:**
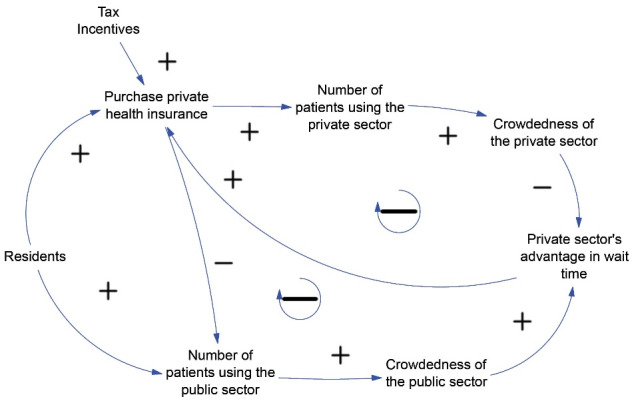
Causal Loop Diagram Showing the Feedback Loop Between Private Insurance Purchase and the Burden of the Private/Public Healthcare System.

 Causal loop diagram is a common way to visualize feedback loops.^33^Figure 1 is a causal loop diagram created in Vensim version 8.2.1 (Ventana Systems, Inc., Harvard, MA, USA). Besides the feedback loop mentioned above, a government initiative to offer tax incentives for purchasing private health insurance positively contributes to its uptake as well.

 We walked through the diagram with five stakeholders recruited from our professional network. They are an international insurance professional, a local policy expert, a biomedical engineer, and two Master of Public Health graduates. There was no objection to the framing of this hypothetical conceptual model.

###  Choice of Modeling Method

 We followed the RIGHT toolkit and the SIMULATE checklist to identify the proper modeling method.^34,35^ System dynamics was selected because it is an approach beneficial for analyzing long-term policies and estimating the time-dependent behavior of a complex system.^18^ It offers the appropriate level of insights for this study’s purposes without the need for an excessive amount of data input. It is a natural choice when a feedback loop is in the conceptual model. A systematic review of system dynamics in healthcare published in 2020 included 253 papers,^36^ showing its increasing popularity since 2013. These papers cover a variety of topics, including patient flow, obesity, workforce demand, and HIV/AIDS.

 “There is an extensive literature describing and categorizing the various steps undertaken as a part of a simulation study.”^37^ The steps are generally very similar in each, with variation in their level of granularity.^37^ One textbook on system dynamics describes the steps as follows: (1) Problem identification; (2) Dynamic hypothesis; (3) Causal Loop Diagram; (4) Stock–Flow Diagram; (5) Parameter estimation; (6) Model validation, sensitivity analysis and policy analysis; (7) Application of the model.^38^ Previous sections of this paper have covered steps one to three. The remaining Methods section covers steps four and five, and the “how to” part of step six. The Results and Discussion sections cover the “results” part of step six, and step seven.

 We introduce the modeling steps of system dynamics here rather than upfront in the paper because our thought process was problem-driven and tool-agnostic. In a textbook on system dynamics, it is usually assumed upfront that a reader wants to build a system dynamic model. In our case, we were driven by the problem and research question, rather than by a particular modeling technique. We were open to any technique that can deepen our understanding of the feedback between health system burden and the demand for health insurance. After analyzing the problem and following the checklists above, we found system dynamics as the most appropriate and natural choice. That is why the specific steps of system dynamics are introduced and followed here. In retrospective, our way of problem identification and hypothesizing is in accordance with system dynamics modeling development process.

###  Stock–Flow Diagram

 Stock–Flow Diagram is usually followed after the causal loop diagram, and is the core feature of system dynamics modeling. It represents the underlying structure of the system. Stock describes the condition or state of the system at any particular time. Flow describe how fast a stock is changing. A stock integrates flows into and out of it, and the net flow into the stock is the rate of change of the stock.^38^ Mathematically, if the quantity of some stock variable at time t is Q(t), then the derivative of Q(t) with respect to t is the flow of changes in the stock. Likewise, the stock at some time t is the integral of the flow from time 0 until time t.^39^ Stock and flow are also known as level and rate.

 In healthcare, system dynamics commonly models patient journey as stocks and flows, where stocks represent steps of the journey, and flows represent patients moving from one step to another.^40^ For example, when modeling an emergency room, triage room can be a stock that accumulates patients “flowing” from walk-in or ambulance. Treatment room can be another stock that accumulates patients flowing from triage.

**Figure 2:**
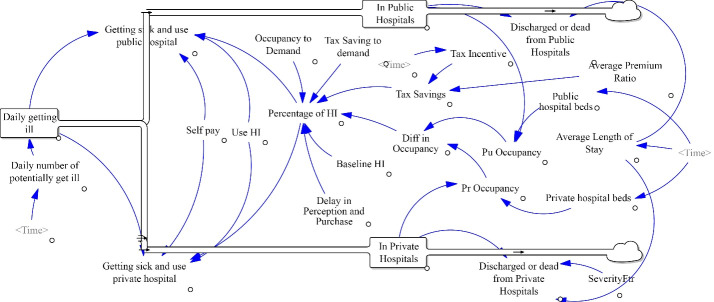
System Dynamics Model Structure Explaining the Feedback Loop Between the Demand for Voluntary Private Insurance and the Burden of the Healthcare System.

 In our patient journey, it starts from a flow of people getting relatively sick who needs hospitalization. The first stock accumulates these people, who then flow into the stocks of either public or private hospitals. After staying in the hospital for a period of time, they flow out of hospital as they get discharged or die. This is shown in Figure 2, which was created in Vensim. The rectangles are stocks, and black arrows are flows. The remaining elements are auxiliary variables. The data sources and equations for stocks, flows and variables are discussed subsequently.

###  Data Sources

 We took data from multiple sources since 2009 as model input in order to produce accurate model output from 2015 to 2019. Data about Hong Kong healthcare services came from the Hong Kong Hospital Authority Statistical Report.^41^ All reports from the 2010-2011 fiscal year onward are publicly available. Between the 2010-2011 and 2016-2017 fiscal years, the number of inpatient discharges or deaths in private institutes is also available. The number of inpatient discharges or deaths in public institutes, as well as the number of beds in both sectors, are available in all reports.

 Data about the Hong Kong health insurance market was obtained from the Thematic Household Survey commissioned by the Census and Statistics Department. Every one to three years, the government commissions a survey on a large sample of households (>10 000) on various topics, including whether they have purchased individual medical insurance.^9^ Although self-reporting bias is unavoidable,^42^ the representativeness of this survey has been acknowledged in academic literature and a briefing to the Legislative Council of Hong Kong.^43,44^ This briefing also provides data on the care-seeking behaviors of those insured. In this paper, we used six Thematic Household Survey reports between 2009 and 2019. More details can be found in Supplementary file 1.

###  Model Equations and Input

 Bed occupancy rate was used as a proxy for crowdedness because it is the only metric available for both the public and private sectors. Other notorious metrics, such as waiting time for outpatient specialists, imaging, chemotherapy, or elective surgeries, are only available for the public sector.

 Since the bed occupancy rate was chosen as the primary feedback driver for health insurance demand, the model focuses on inpatients rather than all patients. The starting point of the model is the number of patients who need inpatient services per year. This is a number we could accurately obtain from the Hospital Authority Statistical Report.

 Depending on other insurance status, a certain percentage of these patients get admitted to public hospitals, and others to private hospitals. To determine the number of patients admitted to public hospitals (PuAdmit) and private hospitals (PrAdmit):


(1) $$PuAdmit=InptNum_{t}.HI_{t}\text{.(}1-UseHI)+InptNum_{t}\text{.}\left(1-HI_{t}\right).\left(1-Selfpay\right)$$

(2) $$PrAdmit=InptNum_{t}.HI_{t}.UseHI+InptNum_{t}\text{.}\left(1-HI_{t}\right).Selfpay$$

where InptNum is the total number of patients who need hospitalization in a year. HI is the percentage of patients with health insurance. UseHI is the percentage of patients with health insurance who will use the insurance benefit to obtain service in the private sector. Therefore, (1-UseHI) refers to percentage of patients, although with health insurance, who will still use the public sector. Selfpay is the percentage of patients without health insurance who are willing to pay for private health services out of pocket.


(3) $$UsingPu_{t}=\int \left(PuAdmit_{t}-PuDischargeOrDeath_{t}\right)dt$$

(4) $$UsingPr_{t}=\int \left(PrAdmit_{t}-PrDischargeOrDeath_{t}\right)dt$$

where UsingPu and UsingPr are the number of patients in the public and private sectors at time *t*. It is the integral of the number of patients admitted minus those discharged or dead over the period *t*. Discharge or death is calculated by dividing the admissions by the average length of stay (LoS), which is PuLoS for the public sector and PrLoS for the private sector. Formulas 3 and 4 represent “stocks” in the terminology of system dynamics modeling and are time-dependent.

 To calculate the bed occupancy rate of public hospitals (PuOccu) and private hospitals (PrOccu) and their difference:


(5) $$PuOccu_{t}=\left(PuAdmit_{t}\;.PuLoS_{t}\right)÷PuBeds_{t}$$

(6) $$PrOccu_{t}=\left(PrAdmit_{t}\;.PuLoS_{t}\;.SeverityFtr\right)÷PrBeds_{t}$$

(7) $$DiffOccu_{t}=PrOccu_{t}-PuOccu_{t}$$

where PuAdmit and PrAdmit refer to the number of patients in public and private hospitals. SeverityFtr is a severity factor that adjusts the average LoS from public hospitals to private hospitals. PuBed and PrBed refer to the number of beds in public and private hospitals.

 Severity factor is needed to attend to the observation that the average LoS in private hospitals is significantly shorter than that in public hospitals (only about 40%) in Hong Kong.^45^ While some proportion of this difference might be due to different treatment protocols or efficiency of private versus public hospitals,^46^ the difference is so large that we believe public hospitals are taking care of sicker patients. Financially, even private insurance might not cover the full cost of treating critical illness in private hospitals, while treatment in public hospitals is almost free for any disease of any treatment duration. Clinically, private hospitals lack some intensive care capabilities, such as the coronary care unit, and might be more risk-averse. One example is hysterectomy. The ratio of abdominal hysterectomies (a more invasive surgery) to laparoscopic hysterectomies (less invasive) is much higher in public hospitals than in private hospitals. Also, the average LoS for abdominal and laparoscopic hysterectomies is both longer in public hospitals than private hospitals.^47-49^

 The core variable of this model is the percentage of patients with health insurance at time step t. It is a result of the feedback effect from the difference in occupancy [Effect(DiffOccu)], and the exogenous effect of a tax incentive [Effect(TaxInct)]. Its calculation depends on whether the tax incentive policy has been implemented.

 If t < the time when tax incentive is implemented,


(8) $$HI_{t}=BaselineHI+Effect\left(DiffOccu_{t}\right)$$

If t ≥ the time when tax incentive is implemented,


(9) $$HI_{t}=BaselineHI+Effect\left(DiffOccu_{t}\right)+Effect\left(TaxInct\right)$$

where BaselineHI is the baseline percentage of patients with individual health insurance. Effect(DiffOccu) denotes the effect on health insurance penetration as a function of the different occupancy rates between the public and private sectors. Based on a review of the literature,^46^ we consider this as an absolute effect, so it is added to the baseline health insurance penetration. Effect(TaxInct) pertains to the effect on health insurance penetration as a function of the tax incentive at time step t when the tax incentive is implemented.


(10) $$Effect\left(DiffOccu_{t}\right)=Delay\left[\left(DiffOccu_{t}-BaselineDiff\right)\;.OccuDmd,\;Duration\right]$$

where Delay is a function to postpone the effect of occupancy rate on health insurance demand, because it takes time for occupancy rate to be published by the government, reported by media, and perceived by consumers either through experience or reading publications/reports. We followed a previous modeling study to assume this “lagged effect” to be one year (eg, variable “Duration” = 1 year).^50^ It is implemented in Vensim version 8.2.1 as the DELAY3 function, which returns a 3rd order exponential delay of the input.^51^ BaselineDiff is the baseline difference between the occupancy rates of public versus private hospitals. DiffOccu is the current difference in occupancy rate between the public and private sector. We focus on the difference rather than the public hospitals’ occupancy alone because the latter will not drive health insurance demand if there is no better private alternative.^6^

 OccuDmd is an elasticity factor in translating changes of occupancy difference into health insurance demand. Because there is no empirical evidence to support a direct translation, this factor needs to concern how occupancy rate of inpatient beds could lead to wait time of inpatient admission, how big the wait time difference is between sectors, and consequently, this difference could lead to people’s demand to purchase private insurance. On the left side of this logical chain, existing research reveals the connection between occupancy rate and the wait time in the Accident & Emergency Department (A&E).^52^ On the right side, there is research to connect the surgery wait time to the demand for insurance.^53^ Assumptions need to be made to bridge these two streams of data in the context of inpatient care. Since in Hong Kong, most of the inpatient admissions in public hospitals are from A&E,^54^ we assumed that the relationship between A&E occupancy and wait time applies to inpatient care in both sectors. This is probably a conservative estimate for the small portion of planned surgeries as well.^55^ OccuDmd is subject to sensitivity analysis.


(11) $$Effect\left(TaxInct\right)=Delay\left[\left(Saving\left(TaxInct\right)÷AvgTax\right)\;.TaxDmd,\;Duration\right]$$

where, similarly, Delay is a function to postpone the effect of a tax incentive (TaxInct) on health insurance demand, because it takes time for people to react to the policy. It is implemented in Vensim version 8.2.1 as the DELAY3 function. Saving(TaxInct) is a function that calculates the tax saving based on a given tax incentive. The result of Saving(TaxInct) divided by AvgTax equals tax saving as an average of income tax (“proportion of tax saved”). TaxDmd is an elasticity factor to translate the proportion of tax saved into health insurance purchases. “Duration” represents the delayed duration of a tax incentive to health insurance demand.

 In accordance with government statistical procedures,^56^ we estimated the average tax per capita to be HK $2876 (US $370).^57,58^ This low value reflects the fact that Hong Kong is a city with extreme income inequality. Over half of the population’s income is below the tax allowance level (HK $132 000, or US $16 980),^59^ and do not pay any tax.


(12) $$Saving\left(TaxInct\right)=TaxInct.\;AvgPrmRatio.\;TaxRate$$

where AvgPrmRatio is the average premium ratio. In the case of Hong Kong, where the tax deduction is high and several tiers of products are available (HK $8000 or US $1029; with Standard Plan and several tiers of Flexi Plans), we assume the average premium is about 85% of the pre-tax deduction, which allows a range of products to meet different needs of consumers and maximizes the tax benefits as much as possible. This ratio is subject to sensitivity analysis. TaxRate pertains to the tax rate bracket applied to the tax reduction. The average tax (HK $2,876) falls into the 6% bracket.^60^ As a result, Saving(TaxInct) = 8000 * 0.85 * 0.06 = HK $408. Saving(TaxInct) ÷ AvgTax = 408 ÷ 2876 = 14.2%.

 Another issue needed to be addressed with model inputs is the “winter surge,” which denotes higher demand for health services during the winter, causing longer waiting time.^61,62^ We divided a 90-day winter surge period into three stages: public/private hospital admission starts linearly increasing 30 days before January 15; the admission reaches the peak and stays at peak surge level during January 15–February 13; the admission linearly decreases back to non-surge level 30 days after February 13. We also derived that the peak level is 1.196 times the non-surge level from the literature.^61^ Let x donate the number of admissions on a non-surge day, from formula: annual admission = (days in a year – 90) * x + 30 * 1.196 * x + 2 * 30 * ((x+1.196 * x /2), we can obtain public/private hospital use and accordingly the occupancy rate for every single day. This is more precise than assuming every day’s admission is the same throughout the year. The model thus becomes closer to reality and has a stronger explanatory power.

 Point estimates for model input (with note and reference) are displayed in Table 1.

**Table 1:** Point Estimates for Model Inputs

**Variable**	**Point Estimate**	**Note **	**Source for the Point Estimate**
InptNum	2009: 4182 (surge) 3496 (non-surge) 2010: 4355 (surge) 3640 (non-surge) 2011: 4469 (surge) 3735 (non-surge) 2012: 4523 (surge) 3780 (non-surge) 2013: 4438 (surge) 3710 (non-surge) 2014: 4569 (surge) 3819 (non-surge) 2015: 4627 (surge) 3867 (non-surge) 2016: 4838 (surge) 4044 (non-surge) 2017: 4893 (surge) 4090 (non-surge) 2018: 4874 (surge) 4074 (non-surge) 2019: 4720 (surge) 3945 (non-surge)	This does not include day patients (those who do not stay overnight in the hospital). Private utilization from 2017 to 2019 is missing from Hospital Authority Statistics Report. Data from Department of Health is used to calculate those. Raw annual inputs are transformed into daily inputs.	^41,63^
UseHI	57%		^44^
Selfpay	6%		^44^
PuLoS (unit: days)	2009: 7.5 2010: 7.5 2011: 7.2 2012: 7.5 2013: 7.4 2014: 7.3 2015: 7.2 2016: 7.1 2017: 7.2 2018: 7.2 2019: 7.5		^41^
PuBeds	2009: 23 046 2010: 23 263 2011: 23 286 2012: 23 378 2013: 23 686 2014: 23 891 2015: 24 161 2016: 24 392 2017: 24 621 2018: 25 155 2019: 25 661	Excluding beds from A&E observation and psychiatry. Numbers of A&E observation beds in 2009, 2017, 2018 and 2019 and psychiatry beds in 2009 are missing and thus made by assumption.	^41^
PrBeds	2009: 4022 2010: 3946 2011: 4098 2012: 4033 2013: 3882 2014: 3906 2015: 4014 2016: 4226 2017: 4644 2018: 4657 2019: 5056	Number of private beds in 2009 is missing and thus made by assumption. Private beds from 2017 to 2019 are from Department of Health. This is the only variable in the model input recorded in the natural year (from January 1 to December 31). Others are all in the fiscal year (from April 1 to March 31 next year).	^41,63^
SeverityFtr	0.4		^64^
BaselineDiff	5.5%	See 2010-2011 Annual Report.	^41^
OccuDmd	2.22*0.6014 = 1.3351	See Table 2 in Cuff et al,^30^ the coefficient of the second dependent variable is (log(p/(1-p))) = 0.4115, which translates to p = 0.6014, where p is the probability of purchasing insurance.	^52,53^
TaxDmd	0.036 ÷ 0.26 = 0.138	Tax incentive as 26% of premium led to 3.6% absolute increase in voluntary individual private health insurance.	^46^
TaxInct	HK $8000 (US $1029)		^24^
AvgTax	HK $2876 (US $370)	2019 Real gross national income (HK $2 988 277 million) minus gross national disposable income ($ 2 966 685 mil) = Total Tax Paid ($21 592 mil). It is then divided by 2019 mid-year population (7 507 400).	^65^
TaxRate	6%		^60^

Abbreviation: A&E, Accident & Emergency.

###  Model Output and Calibration

 The main output of the model is HI, the percentage of the population with voluntary private health insurance. The real value of this indicator is not precise in most healthcare systems where private insurance is voluntary and supplemental in nature.^66^ When comparing our modeling result to the “real” value, we consider a 3% error rate acceptable as per social science guidelines.^67^

 We considered the following three ways of calibration: changing formula (7) to HI = BaselineHI + MAX(0, Effect(DiffOccu)) + Effect(TaxInct); adjusting OccuDmd to avoid demand for insurance being too sensitive to occupancy and improve robustness of the model; experimenting with a reasonable range of Severityftr since no direct evidence for this variable was found. The optimally calibrated result was determined by calculating the mean absolute percentage error (MAPE) for each different combination of parameters/structure and choosing the one with the smallest MAPE for the last 5 years of simulation (2015 to 2019).

###  Sensitivity Analysis

 Multivariate sensitivity simulation was deployed to address uncertainty in several of the constant inputs, and their range estimates are shown in Table 2. Beta distribution was assumed for the first four variables because they are bounded in 0 and 1; normal distribution was assumed for OccuDmd because elasticity is not bounded. For range estimates without specifying citation, we assumed a range of 20% fluctuation centering around the point estimate.

**Table 2:** Range Estimates and Parametrization for Sensitivity Analysis

**Variable **	**Point Estimate**	**Range Estimate**	**Alpha**	**Beta**
UseHI	57%	(51.3%, 62.7%)	171.43	129.324
Selfpay	6%	(5.4%, 6.6%)	375.94	5889.73
TaxDmd	0.036 ÷ 0.26 = 0.138	(0.025 ÷ 0.26, 0.047 ÷ 0.26)^68^	36.547	228.285
SeverityFtr	0.39	(0.351, 0.429)	243.61	381.031
**Variable **	**Point Estimate**	**Range Estimate**	**Mu**	**Sigma**
OccuDmd	0.5	(0.45, 0.55)	0.5	0.025

###  Simulation Setup

 The model is implemented in Vensim version 8.2.1 (Ventana Systems, Inc., Harvard, Massachusetts). The initial time is April 1, 2009, and final time is December 31, 2019. Time step equals 1 day. In sensitivity analysis, 500 simulations were run, and the noise seed is 1234.

###  Reporting 

 We followed reporting guidelines of simulation-based social research.^69,70^ The model is also fully documented using the System Dynamics Model Documentation and Assessment Tool (SDM-Doc).^71^ This tool can automatically analyze a Vensim model and generate complete documentation for every single detail of it.^71^ This file is provided as Supplementary file 2. The executable Vensim.mdl model will be provided to interested readers within 48 hours of request to the corresponding author.

## Results

###  Calibration Result

 The calibration result (details in Supplementary file 3) implies that the greater the OccuDmd, the higher the percentage of HI and the more the simulated curve oscillates; higher SeverityFtr leads to a lower percentage of HI. After rounds of testing, we calibrated SeverityFtr as 0.39 and OccuDmd as 0.5.

###  Simulation Results

 Using the calibrated model, we reproduced the historical patterns of the number of inpatient admissions in the public and private sector, and the percentage of population with health insurance.

**Figure 3:**
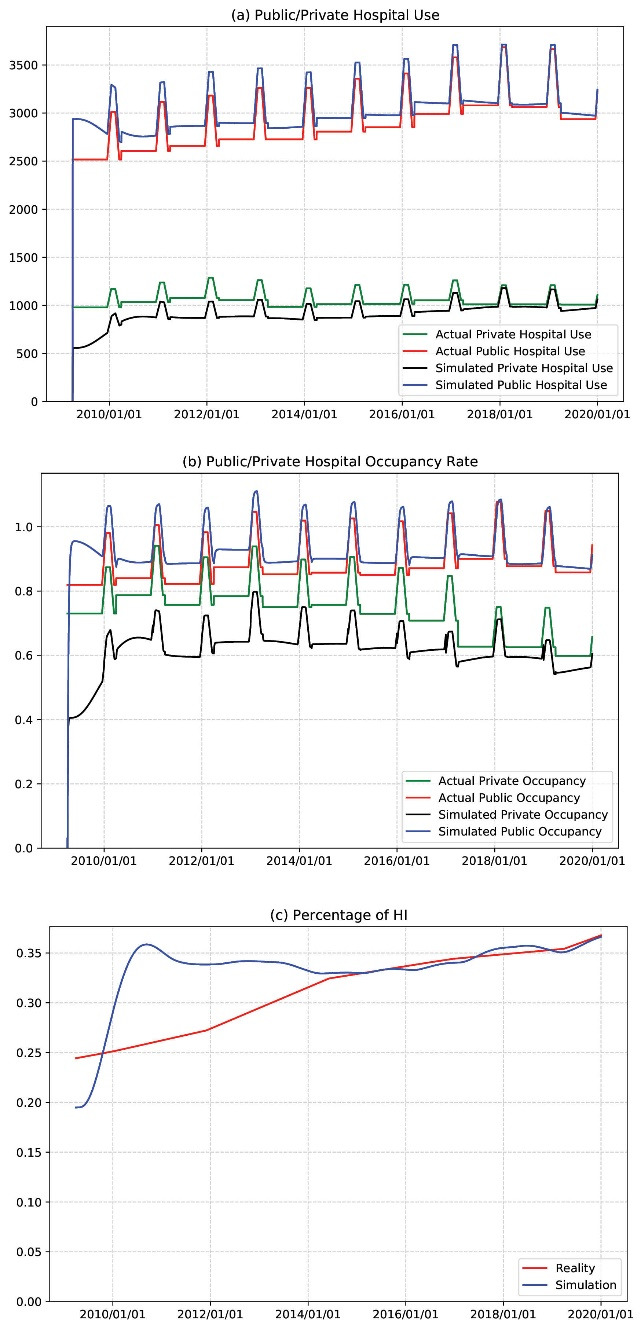
Graphical Result of (a) Simulated Number of Patients Admitted to Private and Public Hospitals vs. Reality; (b) Simulated Occupancy Rate at Private and Public Hospitals vs. Reality; (c) Simulated Percentage of Health Insurance vs. Reality.

 As shown in Figure 3a and 3b, by the end of the simulation (the year 2017 and onward), the results largely reproduce the level and trend of inpatient admissions in both public and private hospitals and the public hospital occupancy rates.

Figure 3c shows the simulation result for the penetration of health insurance. Due to a number of technical and economic reasons elaborated later, the model produces a jump in the first several years as expected. It starts to stabilize in the second half of the time horizon with slight fluctuation and a clear upward-going trend. The last-5-year MAPE is 0.94%, much lower than the acceptable error range of 3%. At the end of 2019, the simulated result is 36.6%, which is very close to the “real value” of 36.7%.

**Figure 4:**
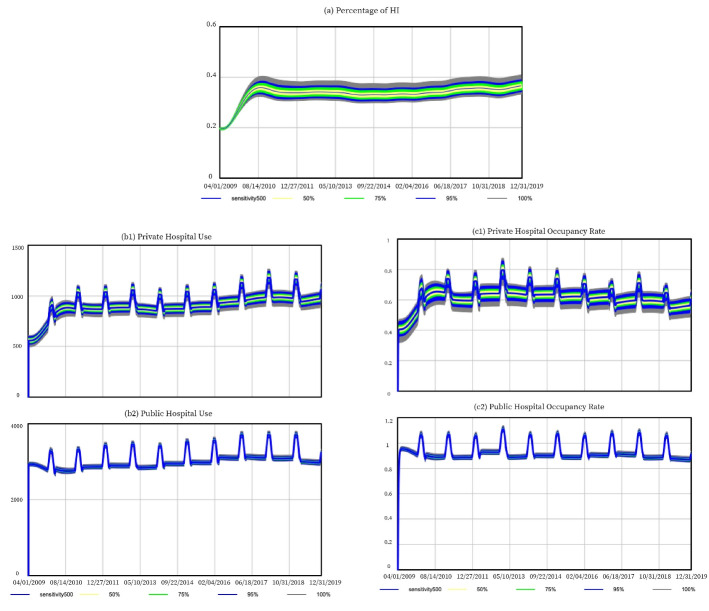
Sensitivity Graph Analysis of (a) Simulated Percentage of Health Insurance; (b) Simulated Number of Patients Admitted to Private and Public Hospitals; (c) Simulated Occupancy Rate at Private and Public Hospitals.

 As shown in Figure 4a, at the end of year 2019, 95% of the simulated percentages of health insurance are between 34.2% and 39.1%, which is only slightly above the 3% error rate from the real value of 36.77%. As shown in Figure 4b and 4c, results on public hospitals are relatively robust toward the uncertainty in model inputs. For private hospitals, the model output is more sensitive.

## Discussion

 What drives people to purchase private health insurance in a society with universal healthcare is an important topic in many countries for academic research, insurance business as well as public policy. In this paper, we built an explanatory model to increase our understanding of the market dynamics between health system burden and insurance demand. It answers a novel research question that takes a temporal and elastic view on the association between health system burden and demand for health insurance. Although the conceptual foundation is intuitive and straightforward, its quantification relies on an extensive literature review and careful calibration. Insights from the present study of the Hong Kong Special Administrative Region of China is transferable to many other countries.

 The present model first quantifies the context of the Hong Kong healthcare system, in which people have free will to choose care providers and pay for services. The public sector takes care of 4 times more hospital admissions that are usually sicker cases (on average 2.6 times longer hospital stays) than the private sector, with its 5 to 6 times more hospital beds. Under such a context, we set the elasticity of intersectoral occupancy difference and the demand for health insurance. Based on literature review and calibration, setting the elasticity as 0.5 can reproduce historical insurance purchasing and care-seeking behaviors from 2015 to 2019. There was a steady increase in the percentage of population with health insurance from June 1, 2014 (model result 32.96% versus real value 32.43%) to April 1, 2019 when VHIS was introduced (35.08% vs. 35.42%), and to December 31, 2019 (36.61% vs. 36.77%). Our model supports the conceptualization that VHIS is an exogenous tax incentive separate from the core feedback mechanism. Our model is relatively robust in sensitivity analysis.

 Soon after the simulation starts, there is a rapid increase in the percentage of health insurance. It is expected because of the “initiation effect” caused by the initial values of the variables and stocks (conceptual entities that accumulate or get depleted). Variables’ initial values might be based on snapshot static values at a particular time point of the system. Stocks’ initial values are usually zeros because they have not accumulated anything. These values might be different from the real values when a system is “live.” Therefore, it is a common practice in simulation modeling to ignore the initial results of the simulation (the “warm up” period).^72,73^ That’s why we configured our model to start from 2009 in order to obtain stable and accurate result from 2015 to 2019.

 With its simple design, this model quantitatively explains a core feedback mechanism, but it inevitably presents several limitations. First, we could only use inpatient bed occupancy as a metric for health system crowdedness because this is the only metric that could be accurately calculated for both public and private sectors in Hong Kong using publicly available data. Also, we made an assumption in the model design that the percentage of people who own and use health insurance is the same between the general public and those who need inpatient services. There is no data on who have insurance among those hospitalized. Since the inpatient admissions we obtained from the government report include all segments of the population, we think this assumption is not unreasonable, and the result from sensitivity analysis seems to support it as well.

## Conclusion

 We answered a novel research question to quantitatively explain the feedback loop between the intersectoral difference in health system burden and the demand for health insurance, a previously overlooked dynamic. With local parameterization, the simple and intuitive structure of this model should be transferable to other universal health systems for better understanding of the system dynamics and more informed policy-making. There is a limit to what tax incentive policy could boost the penetration of health insurance, given the negative feedback loop uncovered and explained in this model. Other accompanying policies are needed to reduce the burden on the public healthcare sector and benefit the entire population.

## Ethical issues

 None, this study uses publicly available secondary data.

## Competing interests

 Authors declare that they have no competing interests.

## Authors’ contributions

 JC contributed to the conception, design, data acquisition, data analysis, manuscript drafting and interpretation of the study. YZ contributed to data acquisition, data analysis, graphics and manuscript drafting. Both authors revised and approved the final manuscript.

## Supplementary files


Supplementary file 1. Reference Data on the Percentage of Population With Individual Voluntary Private Health Insurance.

Supplementary file 2. Full Documentation of the Model Using SDM-Doc.

Supplementary file 3. Graphical Results During Calibration.
