# Supplementary material for: The Feedback Loop Between the Demand for Voluntary Private Insurance and the Burden of Healthcare System: An Explanatory System Dynamics Model of Hong Kong
Source: Int J Health Policy Manag. 2022 Jul 6;11(12):3040–51. doi: 10.34172/ijhpm.2022.6738 (PMC10105189; doi:10.34172/ijhpm.2022.6738)
Supplement: Supplementary file 1 — Reference Data on the Percentage of Population With Individual Voluntary Private Health Insurance. [file ijhpm-11-3040-s001.pdf]

Article title: The Feedback Loop Between the Demand for Voluntary Private Insurance and the Burden of Healthcare System: An Explanatory System Dynamics Model of Hong Kong

Journal name: International Journal of Health Policy and Management (IJHPM)

Authors' information: Yiran Zou[1], Junqiao Chen[2,3,4]*

[1]Center for Data Science, New York University, New York City, NY, USA

[2]Roche Hong Kong and Macau, Hong Kong SAR, China

[3]University Institute of Lisbon (ISCTE-IUL), Lisbon, Portugal

[4]Faculty of Sciences, University of Lisbon, Lisbon, Portugal

(*Corresponding author: junqiao.chen@roche.com)

Supplementary file 1. Reference Data on the Percentage of Population With Individual Voluntary Private Health Insurance

The reference data was obtained from two questions in the Thematic Household Survey. The first question is "Percentage of persons who were entitled to medical benefits provided by employers/companies and/or covered by individually purchased medical insurance among all persons." The second one is "Only entitled to medical benefits provided by employers/companies." Let a denote the result of the first question, and b denote the results of the second question. The percentage of population with individual health insurance is a*(1-b).

A survey was usually conducted over the course of several months. We took the median date as the reference date for each survey. Results of different dates are connected linearly.

Table S1: Percentage of population with individual health insurance from six Thematic Household Surveys

**Report Release Date**	November 2019	December 2017	October 2015	January 2013	December 2010	September 2009
**Survey Date**	November 2018 to February 2019	October 2016 to January 2017	March to August 2014	October 2011 to January 2012	November 2009 to February 2010	February to May 2008
**Convert to an exact date in our model**	12/31/2018	12/1/2016	6/1/2014	12/1/2011	12/31/2009	3/31/2008
**Percentage of population with health insurance**	35.31%	34.39%	32.43%	27.22%	25.11%	23.53%

From 12/31/2018 to 3/31/2019, we assumed the same growth rate as the one from 12/1/2016 to 12/31/2018. This leads to the result of 35.42% (converted to 3/31/2019 as an exact date in our model).

From 4/1/2019 (when Voluntary Health Insurance Scheme was introduced) to 12/31/2019, we assume a 5% annual growth rate based on a Hong Kong Legislative Council Paper No. CB(4)1196/20-21(05) titled "Legislative Council Panel on Health Services: Update on the Voluntary Health Insurance Scheme" (9 July 2021). This leads to the result of 36.77% (converted to 12/31/2019 as an exact date in our model).
