# Supplementary material for: The Feedback Loop Between the Demand for Voluntary Private Insurance and the Burden of Healthcare System: An Explanatory System Dynamics Model of Hong Kong
Source: Int J Health Policy Manag. 2022 Jul 6;11(12):3040–51. doi: 10.34172/ijhpm.2022.6738 (PMC10105189; doi:10.34172/ijhpm.2022.6738)
Supplement: Supplementary file 2 — Full Documentation of the Model Using SDM-Doc. [file ijhpm-11-3040-s002.pdf]

Article title: The Feedback Loop Between the Demand for Voluntary Private Insurance and the Burden of Healthcare System: An Explanatory System Dynamics Model of Hong Kong

Journal name: International Journal of Health Policy and Management (IJHPM)

Authors' information: Yiran Zou[1], Junqiao Chen[2,3,4]*

[1]Center for Data Science, New York University, New York City, NY, USA

[2]Roche Hong Kong and Macau, Hong Kong SAR, China

[3]University Institute of Lisbon (ISCTE-IUL), Lisbon, Portugal

[4]Faculty of Sciences, University of Lisbon, Lisbon, Portugal

(*Corresponding author: junqiao.chen@roche.com)

Supplementary file 2. Full Documentation of the Model Using SDM-Doc

---

Quick Links	All Variables	Variable Link Detail	Variable Types	Views	Groups	Units	Macros	Feedback Loops	Exogenous Variables Analysis	Endogenous Variables Analysis	Link Polarity	View Summary	View-Variable Profile

Model Assessment Results

Model Information	Result
Total Number Of Variables	34|36
Total Number Of State Variables	4 (11.8%)|6 (16.7%)
Total Number Of Stocks	3 (8.8%)|3 (8.3%)
Total Number Of Exogenous Variables	23 (67.6%)|23 (63.9%)
Total Number Of Endogenous Variables	11 (32.4%)|13 (36.1%)
Total Number Of Feedback Loops No IVV (Maximum Loop Length: 5) [2, 5]	6 (1|5|0)
Total Number Of Feedback Loops With IVV (Maximum Loop Length: 5) [0, 0]	0 (0|0|0)

Total Number Of Causal Links	55 (26|15|14)|67 (36|17|14)
Total Number of Rate-to-rate Links	0
Number Of Units Used In The Model (Basic/Combined)	2/1
Total Number Of Equations Using Macros	0 (0.0%)|0 (0.0%)
Variables With Source Information	0 (0.0%)|0 (0.0%)
Dimensionless Unit Variables	0 (0.0%)|0 (0.0%)
Variables without Predefined Min or Max Values	34 (100.0%)|36 (100.0%)
Function Sensitivity Parameters	0 (0.0%)|0 (0.0%)
Data Lookup Tables	0 (0.0%)|0 (0.0%)
Time Unit	Day
Initial Time	733500
Final Time	737426
Reported Time Interval	TIME STEP
Time Step	1
Model Is Fully Formulated	Yes
Model Defined Groups	No

Warnings	Result
Number Of Undocumented Variables	37 (108.8%)|39 (108.3%)
Equations With Embedded Data	5 (14.7%)|7 (19.4%)
Variables Not In Any View	4 (11.8%)|4 (11.1%)

Nonmonotonic Lookup Functions	7 (20.6%)|7 (19.4%)
Cascading Lookup Functions	0 (0.0%)|0 (0.0%)
Non-Zero End Sloped Lookup Functions	1 (2.9%)|1 (2.8%)
Equations With If Then Else Functions	0 (0.0%)|0 (0.0%)
Equations With Min Or Max Functions	0 (0.0%)|0 (0.0%)
Equations With Step Pulse Or Related Functions	0 (0.0%)|0 (0.0%)
Equations With Unit Errors Or Warnings	26 (76.5%)|28 (77.8%)

Potential Omissions	Result
Unused Variables	5 (14.7%)|5 (13.9%)
Supplementary Variables	0 (0.0%)|0 (0.0%)
Supplementary Variables Being Used	0 (0.0%)|0 (0.0%)
Complex Variable	3 (8.8%)|5 (13.9%)
Complex Stock	0 (0.0%)|0 (0.0%)

Variable Types

**L**: Level (3 / **3**)*	**SM**: Smooth (0 / **0**)*	**DE**: Delay (2 / **18**)*†	**LI**: Level Initial (0)	**I**: Initial (0 / **0**)
**C**: Constant (11 / **11**)	**F**: Flow (5 / **5**)	**A**: Auxiliary (20 / **22**)	**Sub**: Subscripts (0)	**D**: Data (0 / **0**)
**G**: Game (0 / **0**)	**T**: Lookup (10 / **10**)*††			

* (State Variables/Total Stocks) † Total Stocks Do Not Include Fixed Delay Variables. †† (Lookup Tables).

Views

View: View 1 (30 Variables)

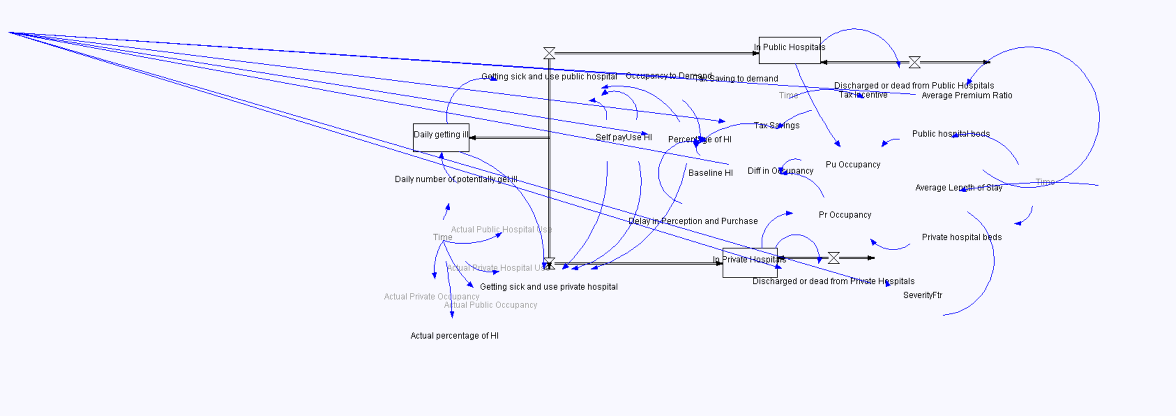

Groups

.Control (4)	Core insurance model 0331 (30)			

**Quick Links:**	A	B	C	D	E	F	G	H	I	J	K	L	M	N	O	P	Q	R	S	T	U	V	W	X	Y	Z

Top	(All) Variables (34 Variables)	
Group	Type	*Variable Name And Description*
Core insurance model 0331	#1 A	**Actual percentage of HI ()**<br>= Actual percentage of HI _SDMlookup(Time)<br>**Present In 1 View:**<br>• View 1<br><br>**Used By**<br>Feedback Loops: 0 (0.0%) (+) 0 [0,0] (-) 0 [0,0]

<table>
<tr><td>Core insurance model 0331</td><td>#3
A</td><td>Actual Private Hospital Use (pt)
= Actual Private Hospital Use _SDMlookup(Time)
Present In 1 View:
View 1
Used By
Feedback Loops: 0 (0.0%) (+) 0 [0,0] (-) 0 [0,0]</td></tr>
<tr><td>Core insurance model 0331</td><td>#5
A</td><td>Actual Private Occupancy ()
= Actual Private Occupancy _SDMlookup(Time)
Present In 1 View:
View 1
Used By
Feedback Loops: 0 (0.0%) (+) 0 [0,0] (-) 0 [0,0]</td></tr>
<tr><td>Core insurance model 0331</td><td>#7
A</td><td>Actual Public Hospital Use (pt)
= Actual Public Hospital Use _SDMlookup(Time)
Present In 1 View:
View 1
Used By
Feedback Loops: 0 (0.0%) (+) 0 [0,0] (-) 0 [0,0]</td></tr>
<tr><td>Core insurance model 0331</td><td>#9
A</td><td>Actual Public Occupancy ()
= Actual Public Occupancy _SDMlookup(Time)
Present In 1 View:
View 1
Used By
Feedback Loops: 0 (0.0%) (+) 0 [0,0] (-) 0 [0,0]</td></tr>
<tr><td>Core insurance model 0331</td><td>#11
A</td><td>Average Length of Stay (Day)
= Average Length of Stay _SDMlookup(Time)
Present In 1 View:
View 1
Used By
Discharged or dead from Private Hospitals
Discharged or dead from Public Hospitals
Feedback Loops: 0 (0.0%) (+) 0 [0,0] (-) 0 [0,0]</td></tr>
</table>

Core insurance model 0331	#12<br>C	**Average Premium Ratio ()**<br>= 0.85<br>**Present In 1 View:**<br>• View 1<br>**Used By**<br>• Tax Savings<br>**Feedback Loops:** 0 (0.0%) (+) 0 [0,0] (-) 0 [0,0]
Core insurance model 0331	#13<br>C	**Baseline HI ()**<br>= 0.222392<br>**Present In 1 View:**<br>• View 1<br>**Used By**<br>• Percentage of HI<br>**Feedback Loops:** 0 (0.0%) (+) 0 [0,0] (-) 0 [0,0]
Core insurance model 0331	#14<br>L	**Daily getting ill (pt)**<br>$= \int$(Daily number of potentially get ill- Getting sick and use private hospital)- Getting sick and use public hospital *dt* + 0.0<br>**Present In 1 View:**<br>• View 1<br>**Used By**<br>• Getting sick and use private hospital<br>• Getting sick and use public hospital<br>**Feedback Loops:** 2 (33.3%) (+) 0 [0,0] (-) 2 [2,2]
Core insurance model 0331	#16<br>F,A	**Daily number of potentially get ill ()**<br>= Daily number of potentially get ill _SDMlookup(Time)<br>**Present In 1 View:**<br>• View 1<br>**Used By**<br>• Daily getting ill<br>**Feedback Loops:** 0 (0.0%) (+) 0 [0,0] (-) 0 [0,0]

Core insurance model 0331	#17<br>C	**Delay in Perception and Purchase (Day)**<br>= 365<br>**Present In 1 View:**<br>• View 1<br>**Used By**<br>• Percentage of HI<br>**Feedback Loops:** 0 (0.0%) (+) 0 [0,0] (-) 0 [0,0]
Core insurance model 0331	#18<br>A	**Diff in Occupancy ()**<br>= Pu Occupancy- Pr Occupancy<br>**Description:** previously: PULSE(2009, 0.125)*0.055 + PULSE(2009.13, 20)*(Pu Occupancy-Pr Occupancy)<br>**Present In 1 View:**<br>• View 1<br>**Used By**<br>• Percentage of HI<br>**Feedback Loops:** 2 (33.3%) (+) 1 [5,5] (-) 1 [5,5]
Core insurance model 0331	#19<br>F,A	**Discharged or dead from Private Hospitals (pt/Day)**<br>= In Private Hospitals/(Average Length of Stay* SeverityFtr)<br>**Present In 1 View:**<br>• View 1<br>**Used By**<br>• In Private Hospitals<br>**Feedback Loops:** 1 (16.7%) (+) 0 [0,0] (-) 1 [2,2]
Core insurance model 0331	#20<br>F,A	**Discharged or dead from Public Hospitals (pt/Day)**<br>= In Public Hospitals/ Average Length of Stay<br>**Present In 1 View:**<br>• View 1<br>**Used By**<br>• In Public Hospitals<br>**Feedback Loops:** 1 (16.7%) (+) 0 [0,0] (-) 1 [2,2]
Core insurance model 0331	#22<br>F,A	**Getting sick and use private hospital ()**<br>= (Daily getting ill* Percentage of HI* Use HI+ Daily getting ill*(1- Percentage of

		HI)* Self pay)/1<br>**Present In 1 View:**<br>• View 1<br>**Used By**<br>• Daily getting ill<br>• In Private Hospitals<br>**Feedback Loops:** 2 (33.3%) (+) 1 [5,5] (-) 1 [2,2]
Core insurance model 0331	#23 F,A	**Getting sick and use public hospital ()**<br>= ((Daily getting ill* Percentage of HI*(1- Use HI)+ Daily getting ill*(1- Percentage of HI)*(1- Self pay)))/1<br>**Present In 1 View:**<br>• View 1<br>**Used By**<br>• Daily getting ill<br>• In Public Hospitals<br>**Feedback Loops:** 2 (33.3%) (+) 0 [0,0] (-) 2 [2,5]
Core insurance model 0331	#24 L	**In Private Hospitals (pt)**<br>= $\int$ Getting sick and use private hospital- Discharged or dead from Private Hospitals *dt* + 0.0<br>**Present In 1 View:**<br>• View 1<br>**Used By**<br>• Discharged or dead from Private Hospitals<br>• Pr Occupancy<br>**Feedback Loops:** 2 (33.3%) (+) 1 [5,5] (-) 1 [2,2]
Core insurance model 0331	#25 L	**In Public Hospitals (pt)**<br>= $\int$ Getting sick and use public hospital- Discharged or dead from Public Hospitals *dt* + 0.0<br>**Present In 1 View:**<br>• View 1<br>**Used By**<br>• Discharged or dead from Public Hospitals

<table>
<tr><td></td><td></td><td>Pu Occupancy
Feedback Loops: 2 (33.3%) (+) 0 [0,0] (-) 2 [2,5]</td></tr>
<tr><td>Core insurance model 0331</td><td>#28
C</td><td>Occupancy to Demand ()
= 0.5
Present In 1 View:
View 1
Used By
Percentage of HI
Feedback Loops: 0 (0.0%) (+) 0 [0,0] (-) 0 [0,0]</td></tr>
<tr><td>Core insurance model 0331</td><td>#29
DE,A</td><td>Percentage of HI ()
= Baseline HI+DELAY3((Diff in Occupancy-0.055)* Occupancy to Demand, Delay in Perception and Purchase)+DELAY3((Tax Savings/2876)* Tax Saving to demand, Delay in Perception and Purchase)
Present In 1 View:
View 1
Used By
Getting sick and use private hospital
Getting sick and use public hospital
Feedback Loops: 2 (33.3%) (+) 1 [5,5] (-) 1 [5,5]</td></tr>
<tr><td>Core insurance model 0331</td><td>#30
A</td><td>Pr Occupancy ()
= In Private Hospitals/ Private hospital beds
Present In 1 View:
View 1
Used By
Diff in Occupancy previously: PULSE(2009, 0.125)*0.055 + PULSE(2009.13, 20)*(Pu Occupancy-Pr Occupancy)
Feedback Loops: 1 (16.7%) (+) 1 [5,5] (-) 0 [0,0]</td></tr>
<tr><td>Core insurance model 0331</td><td>#32
A</td><td>Private hospital beds (pt)
= Private hospital beds _SDMlookup(Time)
Present In 1 View:
View 1
Used By</td></tr>
</table>

		• Pr Occupancy<br><br>**Feedback Loops:** 0 (0.0%) (+) 0 [0,0] (-) 0 [0,0]
Core insurance model 0331	#33 A	**Pu Occupancy ()**<br>= In Public Hospitals/ Public hospital beds<br>**Present In 1 View:**<br><br>• View 1<br><br>**Used By**<br><br>• Diff in Occupancy previously: PULSE(2009, 0.125)*0.055 + PULSE(2009.13, 20)*(Pu Occupancy-Pr Occupancy)<br><br>**Feedback Loops:** 1 (16.7%) (+) 0 [0,0] (-) 1 [5,5]
Core insurance model 0331	#35 A	**Public hospital beds (pt)**<br>= Public hospital beds _SDMlookup(Time)<br>**Description:** 1 bed = 1 pt<br>**Present In 1 View:**<br><br>• View 1<br><br>**Used By**<br><br>• Pu Occupancy<br><br>**Feedback Loops:** 0 (0.0%) (+) 0 [0,0] (-) 0 [0,0]
Core insurance model 0331	#37 C	**Self pay ()**<br>= 0.06<br>**Present In 1 View:**<br><br>• View 1<br><br>**Used By**<br><br>• Getting sick and use private hospital<br>• Getting sick and use public hospital<br><br>**Feedback Loops:** 0 (0.0%) (+) 0 [0,0] (-) 0 [0,0]
Core insurance model 0331	#38 C	**SeverityFtr ()**<br>= 0.39<br>**Present In 1 View:**<br><br>• View 1<br><br>**Used By**

		• Discharged or dead from Private Hospitals<br><br>**Feedback Loops:** 0 (0.0%) (+) 0 [0,0] (-) 0 [0,0]
Core insurance model 0331	#40 A	**Tax Incentive ()**<br>= Tax Incentive _SDMlookup(Time)<br>**Present In 1 View:**<br><br>• View 1<br><br>**Used By**<br><br>• Tax Savings<br><br>**Feedback Loops:** 0 (0.0%) (+) 0 [0,0] (-) 0 [0,0]
Core insurance model 0331	#41 C	**Tax Saving to demand ()**<br>= 0.138<br>**Present In 1 View:**<br><br>• View 1<br><br>**Used By**<br><br>• Percentage of HI<br><br>**Feedback Loops:** 0 (0.0%) (+) 0 [0,0] (-) 0 [0,0]
Core insurance model 0331	#42 A	**Tax Savings ()**<br>= Tax Incentive* Average Premium Ratio*0.06<br>**Present In 1 View:**<br><br>• View 1<br><br>**Used By**<br><br>• Percentage of HI<br><br>**Feedback Loops:** 0 (0.0%) (+) 0 [0,0] (-) 0 [0,0]
Core insurance model 0331	#45 C	**Use HI ()**<br>= 0.57<br>**Present In 1 View:**<br><br>• View 1<br><br>**Used By**<br><br>• Getting sick and use private hospital<br>• Getting sick and use public hospital<br><br>**Feedback Loops:** 0 (0.0%) (+) 0 [0,0] (-) 0 [0,0]

.Control	#21 C	**FINAL TIME** **(Day)**<br>= 737426<br>**Description:** The final time for the simulation.<br>**Present In 0 Views:**<br><br>**Used By**<br><br>**Feedback Loops:** 0 (0.0%) (+) 0 [0,0] (-) 0 [0,0]
.Control	#26 C	**INITIAL TIME** **(Day)**<br>= 733500<br>**Description:** The initial time for the simulation.<br>**Present In 0 Views:**<br><br>**Used By**<br><br>**Feedback Loops:** 0 (0.0%) (+) 0 [0,0] (-) 0 [0,0]
.Control	#36 A	**SAVEPER** **(Day)**<br>= TIME STEP<br>**Description:** The frequency with which output is stored.<br>**Present In 0 Views:**<br><br>**Used By**<br><br>**Feedback Loops:** 0 (0.0%) (+) 0 [0,0] (-) 0 [0,0]
.Control	#43 C	**TIME STEP** **(Day)**<br>= 1<br>**Description:** The time step for the simulation.<br>**Present In 0 Views:**<br><br>**Used By**<br><br>• SAVEPER The frequency with which output is stored.<br><br>**Feedback Loops:** 0 (0.0%) (+) 0 [0,0] (-) 0 [0,0]

(View) Not in View (4 Variables)

Top

(View) Not in View (4 Variables)

Group	Type	*Variable Name And Description*
.Control	#21 C	**FINAL TIME** **(Day)**<br>= 737426<br>**Description:** The final time for the simulation.<br>**Present In 0 Views:**<br><br>**Used By**<br><br>**Feedback Loops:** 0 (0.0%) (+) 0 [0,0] (-) 0 [0,0]

.Control	#26<br>C	**INITIAL TIME (Day)**<br>= 733500<br>**Description:** The initial time for the simulation.<br>**Present In 0 Views:**<br><br>**Used By**<br><br>**Feedback Loops:** 0 (0.0%) (+) 0 [0,0] (-) 0 [0,0]
.Control	#36<br>A	**SAVEPER (Day)**<br>= TIME STEP<br>**Description:** The frequency with which output is stored.<br>**Present In 0 Views:**<br><br>**Used By**<br><br>**Feedback Loops:** 0 (0.0%) (+) 0 [0,0] (-) 0 [0,0]
.Control	#43<br>C	**TIME STEP (Day)**<br>= 1<br>**Description:** The time step for the simulation.<br>**Present In 0 Views:**<br><br>**Used By**<br><br>• SAVEPER The frequency with which output is stored.<br><br>**Feedback Loops:** 0 (0.0%) (+) 0 [0,0] (-) 0 [0,0]

(View) View 1 (30 Variables)

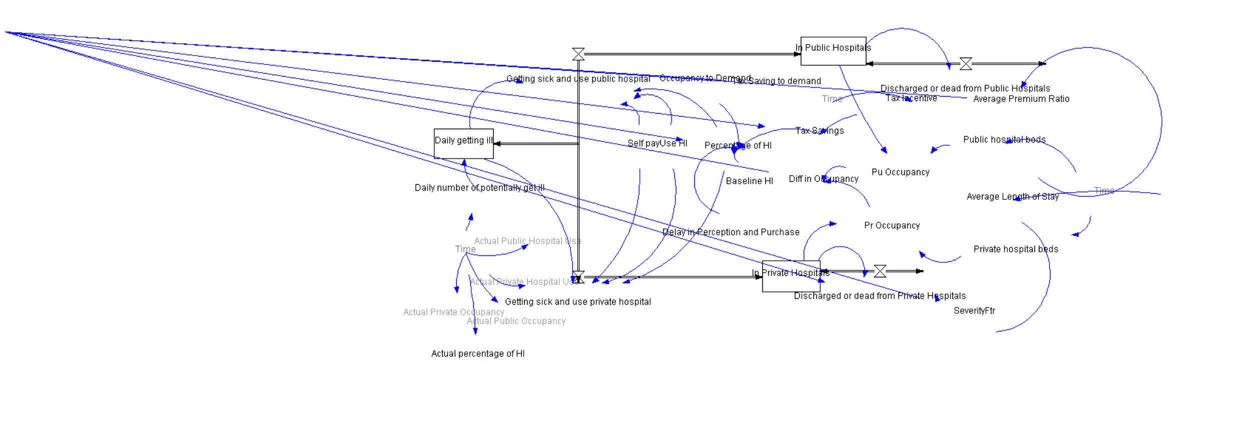

Top	**(View) View 1 (30 Variables)**

Group	Type	*Variable Name And Description*
Core insurance model 0331	#1<br>A	**Actual percentage of HI ()**<br>= Actual percentage of HI _SDMlookup(Time)<br>**Present In 1 View:**

		• View 1<br><br>**Used By**<br>**Feedback Loops:** 0 (0.0%) (+) 0 [0,0] (-) 0 [0,0]
Core insurance model 0331	#3 A	**Actual Private Hospital Use (pt)**<br>= Actual Private Hospital Use _SDMlookup(Time)<br>**Present In 1 View:**<br><br>• View 1<br><br>**Used By**<br>**Feedback Loops:** 0 (0.0%) (+) 0 [0,0] (-) 0 [0,0]
Core insurance model 0331	#5 A	**Actual Private Occupancy ()**<br>= Actual Private Occupancy _SDMlookup(Time)<br>**Present In 1 View:**<br><br>• View 1<br><br>**Used By**<br>**Feedback Loops:** 0 (0.0%) (+) 0 [0,0] (-) 0 [0,0]
Core insurance model 0331	#7 A	**Actual Public Hospital Use (pt)**<br>= Actual Public Hospital Use _SDMlookup(Time)<br>**Present In 1 View:**<br><br>• View 1<br><br>**Used By**<br>**Feedback Loops:** 0 (0.0%) (+) 0 [0,0] (-) 0 [0,0]
Core insurance model 0331	#9 A	**Actual Public Occupancy ()**<br>= Actual Public Occupancy _SDMlookup(Time)<br>**Present In 1 View:**<br><br>• View 1<br><br>**Used By**<br>**Feedback Loops:** 0 (0.0%) (+) 0 [0,0] (-) 0 [0,0]
Core insurance model 0331	#11 A	**Average Length of Stay (Day)**<br>= Average Length of Stay _SDMlookup(Time)<br>**Present In 1 View:**<br><br>• View 1<br><br>**Used By**<br><br>• Discharged or dead from Private Hospitals

		• Discharged or dead from Public Hospitals<br><br>**Feedback Loops:** 0 (0.0%) (+) 0 [0,0] (-) 0 [0,0]
Core insurance model 0331	#12<br>C	**Average Premium Ratio ()**<br>= 0.85<br>**Present In 1 View:**<br><br>• View 1<br><br>**Used By**<br><br>• Tax Savings<br><br>**Feedback Loops:** 0 (0.0%) (+) 0 [0,0] (-) 0 [0,0]
Core insurance model 0331	#13<br>C	**Baseline HI ()**<br>= 0.222392<br>**Present In 1 View:**<br><br>• View 1<br><br>**Used By**<br><br>• Percentage of HI<br><br>**Feedback Loops:** 0 (0.0%) (+) 0 [0,0] (-) 0 [0,0]
Core insurance model 0331	#14<br>L	**Daily getting ill (pt)**<br>= $\int$(Daily number of potentially get ill- Getting sick and use private hospital)- Getting sick and use public hospital *dt* + 0.0<br>**Present In 1 View:**<br><br>• View 1<br><br>**Used By**<br><br>• Getting sick and use private hospital<br>• Getting sick and use public hospital<br><br>**Feedback Loops:** 2 (33.3%) (+) 0 [0,0] (-) 2 [2,2]
Core insurance model 0331	#16<br>F,A	**Daily number of potentially get ill ()**<br>= Daily number of potentially get ill _SDMlookup(Time)<br>**Present In 1 View:**<br><br>• View 1<br><br>**Used By**<br><br>• Daily getting ill

		**Feedback Loops:** 0 (0.0%) (+) 0 [0,0] (-) 0 [0,0]
Core insurance model 0331	#17 C	**Delay in Perception and Purchase (Day)**<br>= 365<br>**Present In 1 View:**<br>• View 1<br>**Used By**<br>• Percentage of HI<br>**Feedback Loops:** 0 (0.0%) (+) 0 [0,0] (-) 0 [0,0]
Core insurance model 0331	#18 A	**Diff in Occupancy ()**<br>= Pu Occupancy- Pr Occupancy<br>**Description:** previously: PULSE(2009, 0.125)*0.055 + PULSE(2009.13, 20)*(Pu Occupancy-Pr Occupancy)<br>**Present In 1 View:**<br>• View 1<br>**Used By**<br>• Percentage of HI<br>**Feedback Loops:** 2 (33.3%) (+) 1 [5,5] (-) 1 [5,5]
Core insurance model 0331	#19 F,A	**Discharged or dead from Private Hospitals (pt/Day)**<br>= In Private Hospitals/(Average Length of Stay* SeverityFtr)<br>**Present In 1 View:**<br>• View 1<br>**Used By**<br>• In Private Hospitals<br>**Feedback Loops:** 1 (16.7%) (+) 0 [0,0] (-) 1 [2,2]
Core insurance model 0331	#20 F,A	**Discharged or dead from Public Hospitals (pt/Day)**<br>= In Public Hospitals/ Average Length of Stay<br>**Present In 1 View:**<br>• View 1<br>**Used By**<br>• In Public Hospitals<br>**Feedback Loops:** 1 (16.7%) (+) 0 [0,0] (-) 1 [2,2]

Core insurance model 0331	#22<br>F,A	**Getting sick and use private hospital ()**<br>= (Daily getting ill* Percentage of HI* Use HI+ Daily getting ill*(1- Percentage of HI)* Self pay)/1<br>**Present In 1 View:**<br>• View 1<br>**Used By**<br>• Daily getting ill<br>• In Private Hospitals<br>**Feedback Loops:** 2 (33.3%) (+) 1 [5,5] (-) 1 [2,2]
Core insurance model 0331	#23<br>F,A	**Getting sick and use public hospital ()**<br>= ((Daily getting ill* Percentage of HI*(1- Use HI)+ Daily getting ill*(1- Percentage of HI)*(1- Self pay)))/1<br>**Present In 1 View:**<br>• View 1<br>**Used By**<br>• Daily getting ill<br>• In Public Hospitals<br>**Feedback Loops:** 2 (33.3%) (+) 0 [0,0] (-) 2 [2,5]
Core insurance model 0331	#24<br>L	**In Private Hospitals (pt)**<br>$= \int$ Getting sick and use private hospital- Discharged or dead from Private Hospitals *dt* + 0.0<br>**Present In 1 View:**<br>• View 1<br>**Used By**<br>• Discharged or dead from Private Hospitals<br>• Pr Occupancy<br>**Feedback Loops:** 2 (33.3%) (+) 1 [5,5] (-) 1 [2,2]
Core insurance model 0331	#25<br>L	**In Public Hospitals (pt)**<br>$= \int$ Getting sick and use public hospital- Discharged or dead from Public Hospitals *dt* + 0.0<br>**Present In 1 View:**<br>• View 1<br>**Used By**

		• Discharged or dead from Public Hospitals<br>• Pu Occupancy<br><br>**Feedback Loops:** 2 (33.3%) (+) 0 [0,0] (-) 2 [2,5]
Core insurance model 0331	#28<br>C	**Occupancy to Demand ()**<br>= 0.5<br>**Present In 1 View:**<br>• View 1<br>**Used By**<br>• Percentage of HI<br><br>**Feedback Loops:** 0 (0.0%) (+) 0 [0,0] (-) 0 [0,0]
Core insurance model 0331	#29<br>DE,A	**Percentage of HI ()**<br>= Baseline HI+DELAY3((Diff in Occupancy-0.055)* Occupancy to Demand, Delay in Perception and Purchase)+DELAY3((Tax Savings/2876)* Tax Saving to demand, Delay in Perception and Purchase)<br>**Present In 1 View:**<br>• View 1<br>**Used By**<br>• Getting sick and use private hospital<br>• Getting sick and use public hospital<br><br>**Feedback Loops:** 2 (33.3%) (+) 1 [5,5] (-) 1 [5,5]
Core insurance model 0331	#30<br>A	**Pr Occupancy ()**<br>= In Private Hospitals/ Private hospital beds<br>**Present In 1 View:**<br>• View 1<br>**Used By**<br>• Diff in Occupancy previously: PULSE(2009, 0.125)*0.055 + PULSE(2009.13, 20)*(Pu Occupancy-Pr Occupancy)<br><br>**Feedback Loops:** 1 (16.7%) (+) 1 [5,5] (-) 0 [0,0]
Core insurance model 0331	#32<br>A	**Private hospital beds (pt)**<br>= Private hospital beds _SDMlookup(Time)<br>**Present In 1 View:**<br>• View 1<br>**Used By**

		• Pr Occupancy<br><br>**Feedback Loops:** 0 (0.0%) (+) 0 [0,0] (-) 0 [0,0]
Core insurance model 0331	#33 A	**Pu Occupancy ()**<br>= In Public Hospitals/ Public hospital beds<br>**Present In 1 View:**<br><br>• View 1<br><br>**Used By**<br><br>• Diff in Occupancy previously: PULSE(2009, 0.125)*0.055 + PULSE(2009.13, 20)*(Pu Occupancy-Pr Occupancy)<br><br>**Feedback Loops:** 1 (16.7%) (+) 0 [0,0] (-) 1 [5,5]
Core insurance model 0331	#35 A	**Public hospital beds (pt)**<br>= Public hospital beds _SDMlookup(Time)<br>**Description:** 1 bed = 1 pt<br>**Present In 1 View:**<br><br>• View 1<br><br>**Used By**<br><br>• Pu Occupancy<br><br>**Feedback Loops:** 0 (0.0%) (+) 0 [0,0] (-) 0 [0,0]
Core insurance model 0331	#37 C	**Self pay ()**<br>= 0.06<br>**Present In 1 View:**<br><br>• View 1<br><br>**Used By**<br><br>• Getting sick and use private hospital<br>• Getting sick and use public hospital<br><br>**Feedback Loops:** 0 (0.0%) (+) 0 [0,0] (-) 0 [0,0]
Core insurance model 0331	#38 C	**SeverityFtr ()**<br>= 0.39<br>**Present In 1 View:**<br><br>• View 1<br><br>**Used By**

		• Discharged or dead from Private Hospitals<br><br>**Feedback Loops:** 0 (0.0%) (+) 0 [0,0] (-) 0 [0,0]
Core insurance model 0331	#40 A	**Tax Incentive ()**<br>= Tax Incentive _SDMlookup(Time)<br>**Present In 1 View:**<br><br>• View 1<br><br>**Used By**<br><br>• Tax Savings<br><br>**Feedback Loops:** 0 (0.0%) (+) 0 [0,0] (-) 0 [0,0]
Core insurance model 0331	#41 C	**Tax Saving to demand ()**<br>= 0.138<br>**Present In 1 View:**<br><br>• View 1<br><br>**Used By**<br><br>• Percentage of HI<br><br>**Feedback Loops:** 0 (0.0%) (+) 0 [0,0] (-) 0 [0,0]
Core insurance model 0331	#42 A	**Tax Savings ()**<br>= Tax Incentive* Average Premium Ratio*0.06<br>**Present In 1 View:**<br><br>• View 1<br><br>**Used By**<br><br>• Percentage of HI<br><br>**Feedback Loops:** 0 (0.0%) (+) 0 [0,0] (-) 0 [0,0]
Core insurance model 0331	#45 C	**Use HI ()**<br>= 0.57<br>**Present In 1 View:**<br><br>• View 1<br><br>**Used By**<br><br>• Getting sick and use private hospital<br>• Getting sick and use public hospital<br><br>**Feedback Loops:** 0 (0.0%) (+) 0 [0,0] (-) 0 [0,0]

Top

(Group) .Control (4 Variables)

Group	Type	*Variable Name And Description*
.Control	#21 C	**FINAL TIME (Day)**<br>= 737426<br>**Description:** The final time for the simulation.<br>**Present In 0 Views:**<br><br>**Used By**<br><br>**Feedback Loops:** 0 (0.0%) (+) 0 [0,0] (-) 0 [0,0]
.Control	#26 C	**INITIAL TIME (Day)**<br>= 733500<br>**Description:** The initial time for the simulation.<br>**Present In 0 Views:**<br><br>**Used By**<br><br>**Feedback Loops:** 0 (0.0%) (+) 0 [0,0] (-) 0 [0,0]
.Control	#36 A	**SAVEPER (Day)**<br>= TIME STEP<br>**Description:** The frequency with which output is stored.<br>**Present In 0 Views:**<br><br>**Used By**<br><br>**Feedback Loops:** 0 (0.0%) (+) 0 [0,0] (-) 0 [0,0]
.Control	#43 C	**TIME STEP (Day)**<br>= 1<br>**Description:** The time step for the simulation.<br>**Present In 0 Views:**<br><br>**Used By**<br><br>• SAVEPER The frequency with which output is stored.<br><br>**Feedback Loops:** 0 (0.0%) (+) 0 [0,0] (-) 0 [0,0]

Top

(Group) Core insurance model 0331 (30 Variables)

Group	Type	*Variable Name And Description*
Core insurance model 0331	#1 A	**Actual percentage of HI ()**<br>= Actual percentage of HI _SDMlookup(Time)<br>**Present In 1 View:**<br><br>• View 1<br><br>**Used By**<br>**Feedback Loops:** 0 (0.0%) (+) 0 [0,0] (-) 0 [0,0]

Core insurance model 0331	#3<br>A	**Actual Private Hospital Use (pt)**<br>= Actual Private Hospital Use _SDMlookup(Time)<br>**Present In 1 View:**<br>• View 1<br>**Used By**<br>**Feedback Loops:** 0 (0.0%) (+) 0 [0,0] (-) 0 [0,0]
Core insurance model 0331	#5<br>A	**Actual Private Occupancy ()**<br>= Actual Private Occupancy _SDMlookup(Time)<br>**Present In 1 View:**<br>• View 1<br>**Used By**<br>**Feedback Loops:** 0 (0.0%) (+) 0 [0,0] (-) 0 [0,0]
Core insurance model 0331	#7<br>A	**Actual Public Hospital Use (pt)**<br>= Actual Public Hospital Use _SDMlookup(Time)<br>**Present In 1 View:**<br>• View 1<br>**Used By**<br>**Feedback Loops:** 0 (0.0%) (+) 0 [0,0] (-) 0 [0,0]
Core insurance model 0331	#9<br>A	**Actual Public Occupancy ()**<br>= Actual Public Occupancy _SDMlookup(Time)<br>**Present In 1 View:**<br>• View 1<br>**Used By**<br>**Feedback Loops:** 0 (0.0%) (+) 0 [0,0] (-) 0 [0,0]
Core insurance model 0331	#11<br>A	**Average Length of Stay (Day)**<br>= Average Length of Stay _SDMlookup(Time)<br>**Present In 1 View:**<br>• View 1<br>**Used By**<br>• Discharged or dead from Private Hospitals<br>• Discharged or dead from Public Hospitals<br>**Feedback Loops:** 0 (0.0%) (+) 0 [0,0] (-) 0 [0,0]

Core insurance model 0331	#12 C	**Average Premium Ratio ()**<br>= 0.85<br>**Present In 1 View:**<br>• View 1<br>**Used By**<br>• Tax Savings<br>**Feedback Loops:** 0 (0.0%) (+) 0 [0,0] (-) 0 [0,0]
Core insurance model 0331	#13 C	**Baseline HI ()**<br>= 0.222392<br>**Present In 1 View:**<br>• View 1<br>**Used By**<br>• Percentage of HI<br>**Feedback Loops:** 0 (0.0%) (+) 0 [0,0] (-) 0 [0,0]
Core insurance model 0331	#14 L	**Daily getting ill (pt)**<br>= $\int$(Daily number of potentially get ill- Getting sick and use private hospital)- Getting sick and use public hospital *dt* + 0.0<br>**Present In 1 View:**<br>• View 1<br>**Used By**<br>• Getting sick and use private hospital<br>• Getting sick and use public hospital<br>**Feedback Loops:** 2 (33.3%) (+) 0 [0,0] (-) 2 [2,2]
Core insurance model 0331	#16 F,A	**Daily number of potentially get ill ()**<br>= Daily number of potentially get ill _SDMlookup(Time)<br>**Present In 1 View:**<br>• View 1<br>**Used By**<br>• Daily getting ill<br>**Feedback Loops:** 0 (0.0%) (+) 0 [0,0] (-) 0 [0,0]

Core insurance model 0331	#17 C	**Delay in Perception and Purchase (Day)**<br>= 365<br>**Present In 1 View:**<br>• View 1<br>**Used By**<br>• Percentage of HI<br>**Feedback Loops:** 0 (0.0%) (+) 0 [0,0] (-) 0 [0,0]
Core insurance model 0331	#18 A	**Diff in Occupancy ()**<br>= Pu Occupancy- Pr Occupancy<br>**Description:** previously: PULSE(2009, 0.125)*0.055 + PULSE(2009.13, 20)*(Pu Occupancy-Pr Occupancy)<br>**Present In 1 View:**<br>• View 1<br>**Used By**<br>• Percentage of HI<br>**Feedback Loops:** 2 (33.3%) (+) 1 [5,5] (-) 1 [5,5]
Core insurance model 0331	#19 F,A	**Discharged or dead from Private Hospitals (pt/Day)**<br>= In Private Hospitals/(Average Length of Stay* SeverityFtr)<br>**Present In 1 View:**<br>• View 1<br>**Used By**<br>• In Private Hospitals<br>**Feedback Loops:** 1 (16.7%) (+) 0 [0,0] (-) 1 [2,2]
Core insurance model 0331	#20 F,A	**Discharged or dead from Public Hospitals (pt/Day)**<br>= In Public Hospitals/ Average Length of Stay<br>**Present In 1 View:**<br>• View 1<br>**Used By**<br>• In Public Hospitals<br>**Feedback Loops:** 1 (16.7%) (+) 0 [0,0] (-) 1 [2,2]
Core insurance model 0331	#22 F,A	**Getting sick and use private hospital ()**<br>= (Daily getting ill* Percentage of HI* Use HI+ Daily getting ill*(1- Percentage of

		HI)* Self pay)/1<br>**Present In 1 View:**<br><br>• View 1<br><br>**Used By**<br><br>• Daily getting ill<br>• In Private Hospitals<br><br>**Feedback Loops:** 2 (33.3%) (+) 1 [5,5] (-) 1 [2,2]
Core insurance model 0331	#23 F,A	**Getting sick and use public hospital ()**<br>= ((Daily getting ill* Percentage of HI*(1- Use HI)+ Daily getting ill*(1- Percentage of HI)*(1- Self pay)))/1<br>**Present In 1 View:**<br><br>• View 1<br><br>**Used By**<br><br>• Daily getting ill<br>• In Public Hospitals<br><br>**Feedback Loops:** 2 (33.3%) (+) 0 [0,0] (-) 2 [2,5]
Core insurance model 0331	#24 L	**In Private Hospitals (pt)**<br>= ∫ Getting sick and use private hospital- Discharged or dead from Private Hospitals *dt* + 0.0<br>**Present In 1 View:**<br><br>• View 1<br><br>**Used By**<br><br>• Discharged or dead from Private Hospitals<br>• Pr Occupancy<br><br>**Feedback Loops:** 2 (33.3%) (+) 1 [5,5] (-) 1 [2,2]
Core insurance model 0331	#25 L	**In Public Hospitals (pt)**<br>= ∫ Getting sick and use public hospital- Discharged or dead from Public Hospitals *dt* + 0.0<br>**Present In 1 View:**<br><br>• View 1<br><br>**Used By**<br><br>• Discharged or dead from Public Hospitals

		• Pu Occupancy<br><br>**Feedback Loops:** 2 (33.3%) (+) 0 [0,0] (-) 2 [2,5]
Core insurance model 0331	#28 C	**Occupancy to Demand ()**<br>= 0.5<br>**Present In 1 View:**<br><br>• View 1<br><br>**Used By**<br><br>• Percentage of HI<br><br>**Feedback Loops:** 0 (0.0%) (+) 0 [0,0] (-) 0 [0,0]
Core insurance model 0331	#29 DE,A	**Percentage of HI ()**<br>= Baseline HI+DELAY3((Diff in Occupancy-0.055)* Occupancy to Demand, Delay in Perception and Purchase)+DELAY3((Tax Savings/2876)* Tax Saving to demand, Delay in Perception and Purchase)<br>**Present In 1 View:**<br><br>• View 1<br><br>**Used By**<br><br>• Getting sick and use private hospital<br>• Getting sick and use public hospital<br><br>**Feedback Loops:** 2 (33.3%) (+) 1 [5,5] (-) 1 [5,5]
Core insurance model 0331	#30 A	**Pr Occupancy ()**<br>= In Private Hospitals/ Private hospital beds<br>**Present In 1 View:**<br><br>• View 1<br><br>**Used By**<br><br>• Diff in Occupancy previously: PULSE(2009, 0.125)*0.055 + PULSE(2009.13, 20)*(Pu Occupancy-Pr Occupancy)<br><br>**Feedback Loops:** 1 (16.7%) (+) 1 [5,5] (-) 0 [0,0]
Core insurance model 0331	#32 A	**Private hospital beds (pt)**<br>= Private hospital beds _SDMlookup(Time)<br>**Present In 1 View:**<br><br>• View 1<br><br>**Used By**

		• Pr Occupancy<br><br>**Feedback Loops:** 0 (0.0%) (+) 0 [0,0] (-) 0 [0,0]
Core insurance model 0331	#33 A	**Pu Occupancy ()**<br>= In Public Hospitals/ Public hospital beds<br>**Present In 1 View:**<br><br>• View 1<br><br>**Used By**<br><br>• Diff in Occupancy previously: PULSE(2009, 0.125)*0.055 + PULSE(2009.13, 20)*(Pu Occupancy-Pr Occupancy)<br><br>**Feedback Loops:** 1 (16.7%) (+) 0 [0,0] (-) 1 [5,5]
Core insurance model 0331	#35 A	**Public hospital beds (pt)**<br>= Public hospital beds _SDMlookup(Time)<br>**Description:** 1 bed = 1 pt<br>**Present In 1 View:**<br><br>• View 1<br><br>**Used By**<br><br>• Pu Occupancy<br><br>**Feedback Loops:** 0 (0.0%) (+) 0 [0,0] (-) 0 [0,0]
Core insurance model 0331	#37 C	**Self pay ()**<br>= 0.06<br>**Present In 1 View:**<br><br>• View 1<br><br>**Used By**<br><br>• Getting sick and use private hospital<br>• Getting sick and use public hospital<br><br>**Feedback Loops:** 0 (0.0%) (+) 0 [0,0] (-) 0 [0,0]
Core insurance model 0331	#38 C	**SeverityFtr ()**<br>= 0.39<br>**Present In 1 View:**<br><br>• View 1<br><br>**Used By**

		• Discharged or dead from Private Hospitals<br><br>**Feedback Loops:** 0 (0.0%) (+) 0 [0,0] (-) 0 [0,0]
Core insurance model 0331	#40 A	**Tax Incentive ()**<br>= Tax Incentive _SDMlookup(Time)<br>**Present In 1 View:**<br><br>• View 1<br><br>**Used By**<br><br>• Tax Savings<br><br>**Feedback Loops:** 0 (0.0%) (+) 0 [0,0] (-) 0 [0,0]
Core insurance model 0331	#41 C	**Tax Saving to demand ()**<br>= 0.138<br>**Present In 1 View:**<br><br>• View 1<br><br>**Used By**<br><br>• Percentage of HI<br><br>**Feedback Loops:** 0 (0.0%) (+) 0 [0,0] (-) 0 [0,0]
Core insurance model 0331	#42 A	**Tax Savings ()**<br>= Tax Incentive* Average Premium Ratio*0.06<br>**Present In 1 View:**<br><br>• View 1<br><br>**Used By**<br><br>• Percentage of HI<br><br>**Feedback Loops:** 0 (0.0%) (+) 0 [0,0] (-) 0 [0,0]
Core insurance model 0331	#45 C	**Use HI ()**<br>= 0.57<br>**Present In 1 View:**<br><br>• View 1<br><br>**Used By**<br><br>• Getting sick and use private hospital<br>• Getting sick and use public hospital<br><br>**Feedback Loops:** 0 (0.0%) (+) 0 [0,0] (-) 0 [0,0]

Top	(Type) Level (3 Variables)

Group	Type	*Variable Name And Description*
Core insurance model 0331	#14 L	**Daily getting ill (pt)**<br>$= \int$ (Daily number of potentially get ill- Getting sick and use private hospital)- Getting sick and use public hospital *dt* + 0.0<br>**Present In 1 View:**<br>• View 1<br>**Used By**<br>• Getting sick and use private hospital<br>• Getting sick and use public hospital<br>**Feedback Loops:** 2 (33.3%) (+) 0 [0,0] (-) 2 [2,2]
Core insurance model 0331	#24 L	**In Private Hospitals (pt)**<br>$= \int$ Getting sick and use private hospital- Discharged or dead from Private Hospitals *dt* + 0.0<br>**Present In 1 View:**<br>• View 1<br>**Used By**<br>• Discharged or dead from Private Hospitals<br>• Pr Occupancy<br>**Feedback Loops:** 2 (33.3%) (+) 1 [5,5] (-) 1 [2,2]
Core insurance model 0331	#25 L	**In Public Hospitals (pt)**<br>$= \int$ Getting sick and use public hospital- Discharged or dead from Public Hospitals *dt* + 0.0<br>**Present In 1 View:**<br>• View 1<br>**Used By**<br>• Discharged or dead from Public Hospitals<br>• Pu Occupancy<br>**Feedback Loops:** 2 (33.3%) (+) 0 [0,0] (-) 2 [2,5]

Top	(Type) Smooth (0 Variables) (0/0)

Group	Type	*Variable Name And Description*

Top

(Type) Delay (1 Variables) (2/18)

Group	Type	*Variable Name And Description*
Core insurance model 0331	#29 DE,A	**Percentage of HI** () = Baseline HI+DELAY3((Diff in Occupancy-0.055)* Occupancy to Demand, Delay in Perception and Purchase)+DELAY3((Tax Savings/2876)* Tax Saving to demand, Delay in Perception and Purchase) **Present In 1 View:** • View 1 **Used By** • Getting sick and use private hospital • Getting sick and use public hospital **Feedback Loops:** 2 (33.3%) (+) 1 [5,5] (-) 1 [5,5]

Top

(Type) Level Initial (0 Variables)

Group	Type	*Variable Name And Description*

Top

(Type) Initial (0 Variables)

Group	Type	*Variable Name And Description*

Top

(Type) Constant (11 Variables)

Group	Type	*Variable Name And Description*
Core insurance model 0331	#12 C	**Average Premium Ratio** () = 0.85 **Present In 1 View:** • View 1 **Used By** • Tax Savings **Feedback Loops:** 0 (0.0%) (+) 0 [0,0] (-) 0 [0,0]
Core insurance model 0331	#13 C	**Baseline HI** () = 0.222392 **Present In 1 View:**

		• View 1<br><br>**Used By**<br><br>• Percentage of HI<br><br>**Feedback Loops:** 0 (0.0%) (+) 0 [0,0] (-) 0 [0,0]
Core insurance model 0331	#17<br>C	**Delay in Perception and Purchase (Day)**<br>= 365<br>**Present In 1 View:**<br><br>• View 1<br><br>**Used By**<br><br>• Percentage of HI<br><br>**Feedback Loops:** 0 (0.0%) (+) 0 [0,0] (-) 0 [0,0]
Core insurance model 0331	#28<br>C	**Occupancy to Demand ()**<br>= 0.5<br>**Present In 1 View:**<br><br>• View 1<br><br>**Used By**<br><br>• Percentage of HI<br><br>**Feedback Loops:** 0 (0.0%) (+) 0 [0,0] (-) 0 [0,0]
Core insurance model 0331	#37<br>C	**Self pay ()**<br>= 0.06<br>**Present In 1 View:**<br><br>• View 1<br><br>**Used By**<br><br>• Getting sick and use private hospital<br>• Getting sick and use public hospital<br><br>**Feedback Loops:** 0 (0.0%) (+) 0 [0,0] (-) 0 [0,0]
Core insurance model 0331	#38<br>C	**SeverityFtr ()**<br>= 0.39<br>**Present In 1 View:**<br><br>• View 1<br><br>**Used By**

		• Discharged or dead from Private Hospitals<br><br>**Feedback Loops:** 0 (0.0%) (+) 0 [0,0] (-) 0 [0,0]
Core insurance model 0331	#41<br>C	**Tax Saving to demand ()**<br>= 0.138<br>**Present In 1 View:**<br><br>• View 1<br><br>**Used By**<br><br>• Percentage of HI<br><br>**Feedback Loops:** 0 (0.0%) (+) 0 [0,0] (-) 0 [0,0]
Core insurance model 0331	#45<br>C	**Use HI ()**<br>= 0.57<br>**Present In 1 View:**<br><br>• View 1<br><br>**Used By**<br><br>• Getting sick and use private hospital<br>• Getting sick and use public hospital<br><br>**Feedback Loops:** 0 (0.0%) (+) 0 [0,0] (-) 0 [0,0]
.Control	#21<br>C	**FINAL TIME (Day)**<br>= 737426<br>**Description:** The final time for the simulation.<br>**Present In 0 Views:**<br><br>**Used By**<br><br>**Feedback Loops:** 0 (0.0%) (+) 0 [0,0] (-) 0 [0,0]
.Control	#26<br>C	**INITIAL TIME (Day)**<br>= 733500<br>**Description:** The initial time for the simulation.<br>**Present In 0 Views:**<br><br>**Used By**<br><br>**Feedback Loops:** 0 (0.0%) (+) 0 [0,0] (-) 0 [0,0]
.Control	#43<br>C	**TIME STEP (Day)**<br>= 1<br>**Description:** The time step for the simulation.<br>**Present In 0 Views:**<br><br>**Used By**

		• SAVEPER The frequency with which output is stored.<br><br>Feedback Loops: 0 (0.0%) (+) 0 [0,0] (-) 0 [0,0]

Top	(Type) Flow (5 Variables)

Group	Type	*Variable Name And Description*
Core insurance model 0331	#16 F,A	**Daily number of potentially get ill ()**<br>= Daily number of potentially get ill _SDMlookup(Time)<br>**Present In 1 View:**<br>• View 1<br>**Used By**<br>• Daily getting ill<br>Feedback Loops: 0 (0.0%) (+) 0 [0,0] (-) 0 [0,0]
Core insurance model 0331	#19 F,A	**Discharged or dead from Private Hospitals (pt/Day)**<br>= In Private Hospitals/(Average Length of Stay* SeverityFtr)<br>**Present In 1 View:**<br>• View 1<br>**Used By**<br>• In Private Hospitals<br>Feedback Loops: 1 (16.7%) (+) 0 [0,0] (-) 1 [2,2]
Core insurance model 0331	#20 F,A	**Discharged or dead from Public Hospitals (pt/Day)**<br>= In Public Hospitals/ Average Length of Stay<br>**Present In 1 View:**<br>• View 1<br>**Used By**<br>• In Public Hospitals<br>Feedback Loops: 1 (16.7%) (+) 0 [0,0] (-) 1 [2,2]
Core insurance model 0331	#22 F,A	**Getting sick and use private hospital ()**<br>= (Daily getting ill* Percentage of HI* Use HI+ Daily getting ill*(1- Percentage of HI)* Self pay)/1<br>**Present In 1 View:**<br>• View 1<br>**Used By**

		• Daily getting ill<br>• In Private Hospitals<br><br>**Feedback Loops:** 2 (33.3%) (+) 1 [5,5] (-) 1 [2,2]
Core insurance model 0331	#23<br>F,A	**Getting sick and use public hospital ()**<br>= ((Daily getting ill* Percentage of HI*(1- Use HI)+ Daily getting ill*(1- Percentage of HI)*(1- Self pay)))/1<br>**Present In 1 View:**<br><br>• View 1<br><br>**Used By**<br><br>• Daily getting ill<br>• In Public Hospitals<br><br>**Feedback Loops:** 2 (33.3%) (+) 0 [0,0] (-) 2 [2,5]

Top	(Type) Auxiliary (20 Variables)

Group	Type	*Variable Name And Description*
Core insurance model 0331	#1<br>A	**Actual percentage of HI ()**<br>= Actual percentage of HI _SDMlookup(Time)<br>**Present In 1 View:**<br><br>• View 1<br><br>**Used By**<br>**Feedback Loops:** 0 (0.0%) (+) 0 [0,0] (-) 0 [0,0]
Core insurance model 0331	#3<br>A	**Actual Private Hospital Use (pt)**<br>= Actual Private Hospital Use _SDMlookup(Time)<br>**Present In 1 View:**<br><br>• View 1<br><br>**Used By**<br>**Feedback Loops:** 0 (0.0%) (+) 0 [0,0] (-) 0 [0,0]
Core insurance model 0331	#5<br>A	**Actual Private Occupancy ()**<br>= Actual Private Occupancy _SDMlookup(Time)<br>**Present In 1 View:**<br><br>• View 1<br><br>**Used By**<br>**Feedback Loops:** 0 (0.0%) (+) 0 [0,0] (-) 0 [0,0]

Core insurance model 0331	#7<br>A	**Actual Public Hospital Use (pt)**<br>= Actual Public Hospital Use _SDMlookup(Time)<br>**Present In 1 View:**<br>• View 1<br>**Used By**<br>**Feedback Loops:** 0 (0.0%) (+) 0 [0,0] (-) 0 [0,0]
Core insurance model 0331	#9<br>A	**Actual Public Occupancy ()**<br>= Actual Public Occupancy _SDMlookup(Time)<br>**Present In 1 View:**<br>• View 1<br>**Used By**<br>**Feedback Loops:** 0 (0.0%) (+) 0 [0,0] (-) 0 [0,0]
Core insurance model 0331	#11<br>A	**Average Length of Stay (Day)**<br>= Average Length of Stay _SDMlookup(Time)<br>**Present In 1 View:**<br>• View 1<br>**Used By**<br>• Discharged or dead from Private Hospitals<br>• Discharged or dead from Public Hospitals<br>**Feedback Loops:** 0 (0.0%) (+) 0 [0,0] (-) 0 [0,0]
Core insurance model 0331	#16<br>F,A	**Daily number of potentially get ill ()**<br>= Daily number of potentially get ill _SDMlookup(Time)<br>**Present In 1 View:**<br>• View 1<br>**Used By**<br>• Daily getting ill<br>**Feedback Loops:** 0 (0.0%) (+) 0 [0,0] (-) 0 [0,0]
Core insurance model 0331	#18<br>A	**Diff in Occupancy ()**<br>= Pu Occupancy- Pr Occupancy<br>**Description:** previously: PULSE(2009, 0.125)*0.055 + PULSE(2009.13, 20)*(Pu Occupancy-Pr Occupancy)<br>**Present In 1 View:**<br>• View 1<br>**Used By**

		• Percentage of HI<br><br>**Feedback Loops:** 2 (33.3%) (+) 1 [5,5] (-) 1 [5,5]
Core insurance model 0331	#19 F,A	**Discharged or dead from Private Hospitals (pt/Day)**<br>= In Private Hospitals/(Average Length of Stay* SeverityFtr)<br>**Present In 1 View:**<br><br>• View 1<br><br>**Used By**<br><br>• In Private Hospitals<br><br>**Feedback Loops:** 1 (16.7%) (+) 0 [0,0] (-) 1 [2,2]
Core insurance model 0331	#20 F,A	**Discharged or dead from Public Hospitals (pt/Day)**<br>= In Public Hospitals/ Average Length of Stay<br>**Present In 1 View:**<br><br>• View 1<br><br>**Used By**<br><br>• In Public Hospitals<br><br>**Feedback Loops:** 1 (16.7%) (+) 0 [0,0] (-) 1 [2,2]
Core insurance model 0331	#22 F,A	**Getting sick and use private hospital ()**<br>= (Daily getting ill* Percentage of HI* Use HI+ Daily getting ill*(1- Percentage of HI)* Self pay)/1<br>**Present In 1 View:**<br><br>• View 1<br><br>**Used By**<br><br>• Daily getting ill<br>• In Private Hospitals<br><br>**Feedback Loops:** 2 (33.3%) (+) 1 [5,5] (-) 1 [2,2]
Core insurance model 0331	#23 F,A	**Getting sick and use public hospital ()**<br>= ((Daily getting ill* Percentage of HI*(1- Use HI)+ Daily getting ill*(1- Percentage of HI)*(1- Self pay)))/1<br>**Present In 1 View:**<br><br>• View 1<br><br>**Used By**<br><br>• Daily getting ill

<table>
<tr><td></td><td></td><td>In Public Hospitals
Feedback Loops: 2 (33.3%) (+) 0 [0,0] (-) 2 [2,5]</td></tr>
<tr><td>Core insurance model 0331</td><td>#29 DE,A</td><td>Percentage of HI ()
= Baseline HI+DELAY3((Diff in Occupancy-0.055)* Occupancy to Demand, Delay in Perception and Purchase)+DELAY3((Tax Savings/2876)* Tax Saving to demand, Delay in Perception and Purchase)
Present In 1 View:
View 1
Used By
Getting sick and use private hospital
Getting sick and use public hospital
Feedback Loops: 2 (33.3%) (+) 1 [5,5] (-) 1 [5,5]</td></tr>
<tr><td>Core insurance model 0331</td><td>#30 A</td><td>Pr Occupancy ()
= In Private Hospitals/ Private hospital beds
Present In 1 View:
View 1
Used By
Diff in Occupancy previously: PULSE(2009, 0.125)*0.055 + PULSE(2009.13, 20)*(Pu Occupancy-Pr Occupancy)
Feedback Loops: 1 (16.7%) (+) 1 [5,5] (-) 0 [0,0]</td></tr>
<tr><td>Core insurance model 0331</td><td>#32 A</td><td>Private hospital beds (pt)
= Private hospital beds _SDMlookup(Time)
Present In 1 View:
View 1
Used By
Pr Occupancy
Feedback Loops: 0 (0.0%) (+) 0 [0,0] (-) 0 [0,0]</td></tr>
<tr><td>Core insurance model 0331</td><td>#33 A</td><td>Pu Occupancy ()
= In Public Hospitals/ Public hospital beds
Present In 1 View:
View 1
Used By</td></tr>
</table>

		• Diff in Occupancy previously: PULSE(2009, 0.125)*0.055 + PULSE(2009.13, 20)*(Pu Occupancy-Pr Occupancy)<br><br>**Feedback Loops:** 1 (16.7%) (+) 0 [0,0] (-) 1 [5,5]
Core insurance model 0331	#35 A	**Public hospital beds (pt)**<br>= Public hospital beds _SDMlookup(Time)<br>**Description:** 1 bed = 1 pt<br>**Present In 1 View:**<br>• View 1<br>**Used By**<br>• Pu Occupancy<br>**Feedback Loops:** 0 (0.0%) (+) 0 [0,0] (-) 0 [0,0]
Core insurance model 0331	#40 A	**Tax Incentive ()**<br>= Tax Incentive _SDMlookup(Time)<br>**Present In 1 View:**<br>• View 1<br>**Used By**<br>• Tax Savings<br>**Feedback Loops:** 0 (0.0%) (+) 0 [0,0] (-) 0 [0,0]
Core insurance model 0331	#42 A	**Tax Savings ()**<br>= Tax Incentive* Average Premium Ratio*0.06<br>**Present In 1 View:**<br>• View 1<br>**Used By**<br>• Percentage of HI<br>**Feedback Loops:** 0 (0.0%) (+) 0 [0,0] (-) 0 [0,0]
.Control	#36 A	**SAVEPER (Day)**<br>= TIME STEP<br>**Description:** The frequency with which output is stored.<br>**Present In 0 Views:**<br>**Used By**<br>**Feedback Loops:** 0 (0.0%) (+) 0 [0,0] (-) 0 [0,0]

Top

(Type) Subscripts (0 Variables)

Group	Type	*Variable Name And Description*

Top	# (Type) Data (0 Variables)

Group	Type	*Variable Name And Description*

Top	# (Type) Game (0 Variables)

Group	Type	*Variable Name And Description*

Top	# (Type) Lookup (10 Variables)

Group	Type	*Variable Name And Description*
Core insurance model 0331	#0 A, T	**Actual percentage of HI _SDMlookup** () Actual percentage of HI _SDMlookup([(733500,0)-(737517,1)],(733134,0.2353),(733774,0.2511),(734474,0.2722),(735387,0.3243),(736301,0.3439),(737061,0.3531),(737151,0.3542),(737426,0.3677)) 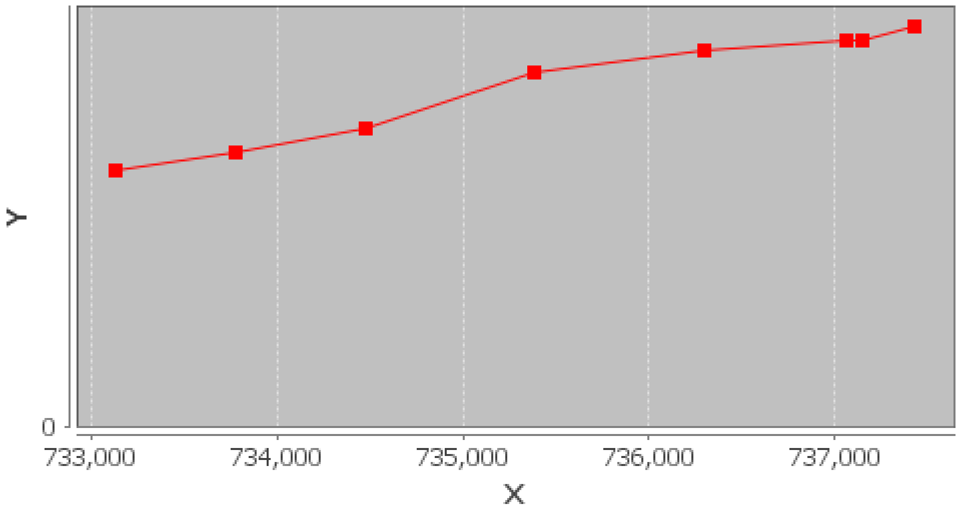 **Present In 0 Views:** **Used By** • Actual percentage of HI **Feedback Loops:** 0 (0.0%) (+) 0 [0,0] (-) 0 [0,0]
Core insura	#2 A, T	**Actual Private Hospital Use _SDMlookup** (pt) Actual Private Hospital Use _SDMlookup([(733500,0)-(737517,10000)],(733500,979),(733759,979),(733789,1171),(733819,1171),(733849,979),(733864,979),(733865,1035),(734124,1035),(734154,1238),(734184,1238),(734214,1035),(734229,1035),(734230,1077),(734489,1077),(734519,1288),(734549,1288),(734579,1077),(734595,1077),(734596,1055),(734855,1055),(734885,1262),(734915,1262),(734945,1055),(734960,1055),(734961,984),(735220,984),(735250,1177

<table>
<tr>
<td>nc
e
m
od
el
03
31</td>
<td></td>
<td>),(735280,1177),(735310,984),(735325,984),(735326,1013),(735585,1013),(735615,1212),(735645,1212),(735675,1013),(735690,1013),(735691,1016),(735950,1016),(735980,1215),(736010,1215),(736040,1016),(736056,1016),(736057,1053),(736316,1053),(736346,1260),(736376,1260),(736406,1053),(736421,1053),(736422,1011),(736681,1011),(736711,1209),(736741,1209),(736771,1011),(736786,1011),(736787,1011),(737046,1011),(737076,1210),(737106,1210),(737136,1011),(737151,1011),(737152,1008),(737411,1008),(737441,1206),(737471,1206),(737501,1008),(737517,1008))
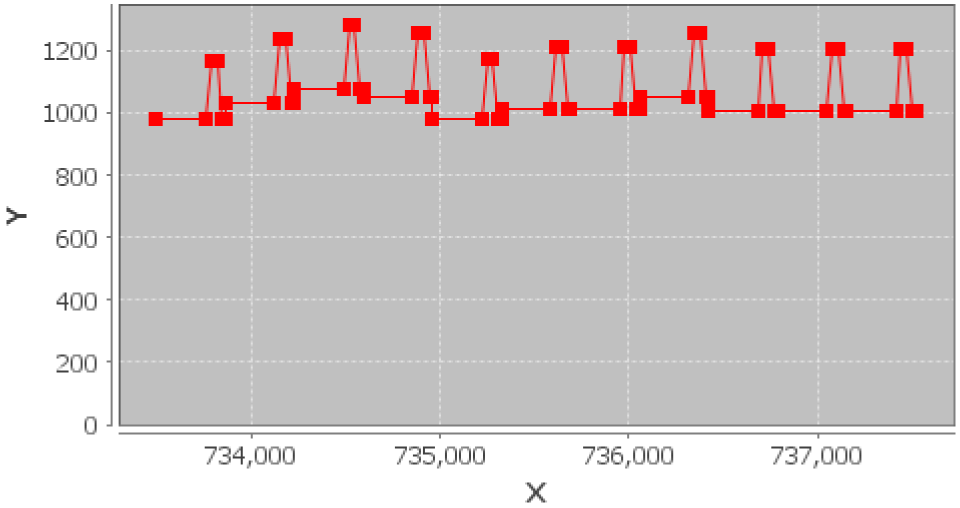

Present In 0 Views:
Used By
Actual Private Hospital Use
Feedback Loops: 0 (0.0%) (+) 0 [0,0] (-) 0 [0,0]</td>
</tr>
<tr>
<td>C
or
e
in
su
ra
nc
e
m
od
el
03
31</td>
<td>#
4
A
,
T</td>
<td>Actual Private Occupancy _SDMlookup ()
Actual Private Occupancy _SDMlookup([(733500,0)-(737157,2)],(733500,0.73),(733759,0.73),(733789,0.874),(733819,0.874),(733849,0.73),(733864,0.73),(733865,0.787),(734124,0.787),(734154,0.941),(734184,0.941),(734214,0.787),(734229,0.787),(734230,0.757),(734489,0.757),(734519,0.905),(734549,0.905),(734579,0.757),(734595,0.757),(734596,0.784),(734855,0.784),(734885,0.939),(734915,0.939),(734945,0.784),(734960,0.784),(734961,0.75),(735220,0.75),(735250,0.898),(735280,0.898),(735310,0.75),(735325,0.75),(735326,0.757),(735585,0.757),(735615,0.906),(735645,0.906),(735675,0.757),(735690,0.757),(735691,0.729),(735950,0.729),(735980,0.872),(736010,0.872),(736040,0.729),(736056,0.729),(736057,0.708),(736316,0.708),(736346,0.847),(736376,0.847),(736406,0.708),(736421,0.708),(736422,0.627),(736681,0.627),(736711,0.75),(736741,0.75),(736771,0.627),(736786,0.627),(736787,0.625),(737046,0.625),(737076,0.748),(737106,0.748),(737136,0.625),(737151,0.625),(737152,0.598),(737411,0.598),(737441,0.716),(737471,0.716),(737501,0.598),(737517,0.598))</td>
</tr>
</table>

<table>
<tr><td></td><td></td><td>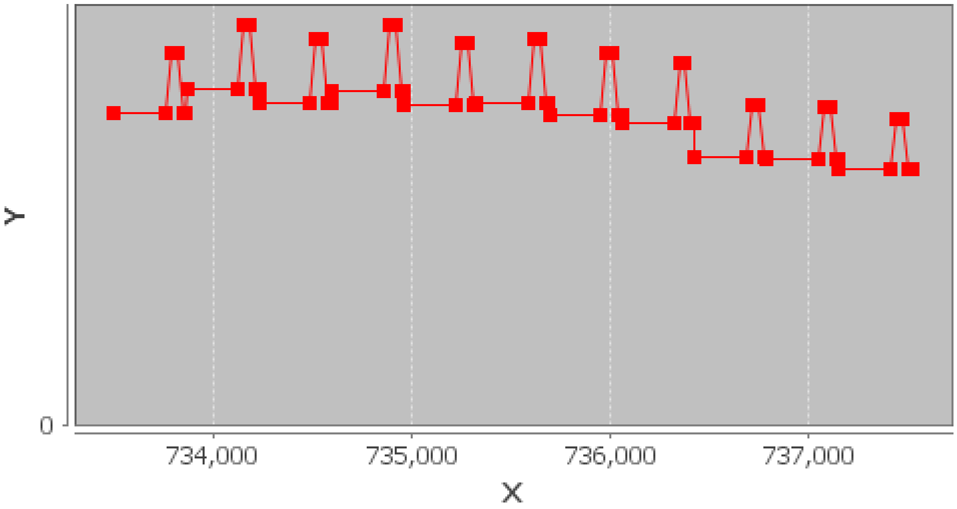
Present In 0 Views:

Used By
Actual Private Occupancy
Feedback Loops: 0 (0.0%) (+) 0 [0,0] (-) 0 [0,0]</td></tr>
<tr><td>Core insurance model 0331</td><td>#6 A, T</td><td>Actual Public Hospital Use _SDMlookup (pt)
Actual Public Hospital Use _SDMlookup([(733500,0)-(737517,10000)],(733500,2517),(733759,2517),(733789,3011),(733819,3011),(733849,2517),(733864,2517),(733865,2605),(734124,2605),(734154,3117),(734184,3117),(734214,2605),(734229,2605),(734230,2658),(734489,2658),(734519,3181),(734549,3181),(734579,2658),(734595,2658),(734596,2726),(734855,2726),(734885,3261),(734915,3261),(734945,2726),(734960,2726),(734961,2726),(735220,2726),(735250,3261),(735280,3261),(735310,2726),(735325,2726),(735326,2806),(735585,2806),(735615,3357),(735645,3357),(735675,2806),(735690,2806),(735691,2852),(735950,2852),(735980,3412),(736010,3412),(736040,2852),(736056,2852),(736057,2991),(736316,2991),(736346,3578),(736376,3578),(736406,2991),(736421,2991),(736422,3079),(736681,3079),(736711,3684),(736741,3684),(736771,3079),(736786,3079),(736787,3062),(737046,3062),(737076,3664),(737106,3664),(737136,3062),(737151,3062),(737152,2936),(737411,2936),(737441,3513),(737471,3513),(737501,2936),(737517,2936))</td></tr>
</table>

		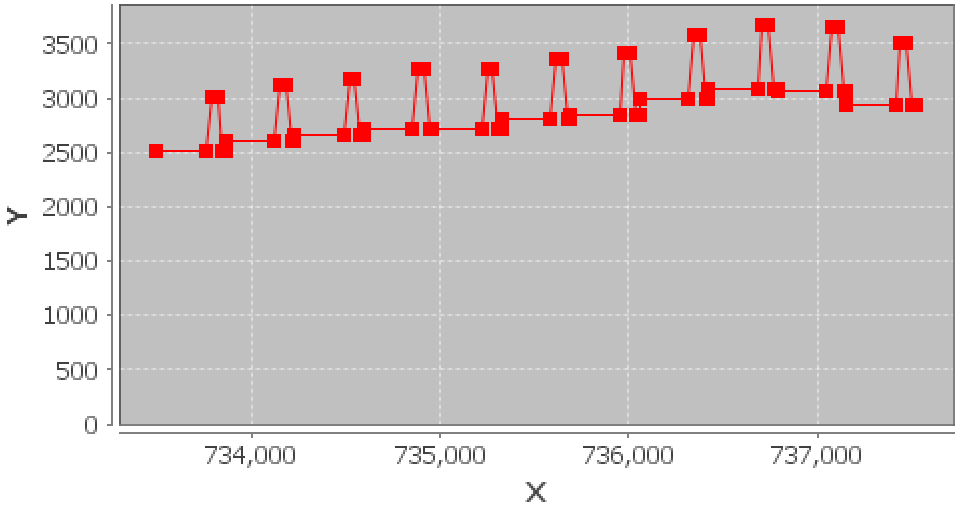<br><br>**Present In 0 Views:**<br>**Used By**<br>• Actual Public Hospital Use<br>**Feedback Loops:** 0 (0.0%) (+) 0 [0,0] (-) 0 [0,0]
Core insurance model 0331	#8A,T	**Actual Public Occupancy _SDMlookup ()**<br>Actual Public Occupancy _SDMlookup([(733500,0)-(737157,2)],(733500,0.819),(733759,0.819),(733789,0.98),(733819,0.98),(733849,0.819),(733864,0.819),(733865,0.84),(734124,0.84),(734154,1.005),(734184,1.005),(734214,0.84),(734229,0.84),(734230,0.822),(734489,0.822),(734519,0.983),(734549,0.983),(734579,0.822),(734595,0.822),(734596,0.874),(734855,0.874),(734885,1.046),(734915,1.046),(734945,0.874),(734960,0.874),(734961,0.852),(735220,0.852),(735250,1.019),(735280,1.019),(735310,0.852),(735325,0.852),(735326,0.857),(735585,0.857),(735615,1.026),(735645,1.026),(735675,0.857),(735690,0.857),(735691,0.85),(735950,0.85),(735980,1.017),(736010,1.017),(736040,0.85),(736056,0.85),(736057,0.871),(736316,0.871),(736346,1.042),(736376,1.042),(736406,0.871),(736421,0.871),(736422,0.9),(736681,0.9),(736711,1.077),(736741,1.077),(736771,0.9),(736786,0.9),(736787,0.877),(737046,0.877),(737076,1.049),(737106,1.049),(737136,0.877),(737151,0.877),(737152,0.858),(737411,0.858),(737441,1.027),(737471,1.027),(737501,0.858),(737517,0.858))

		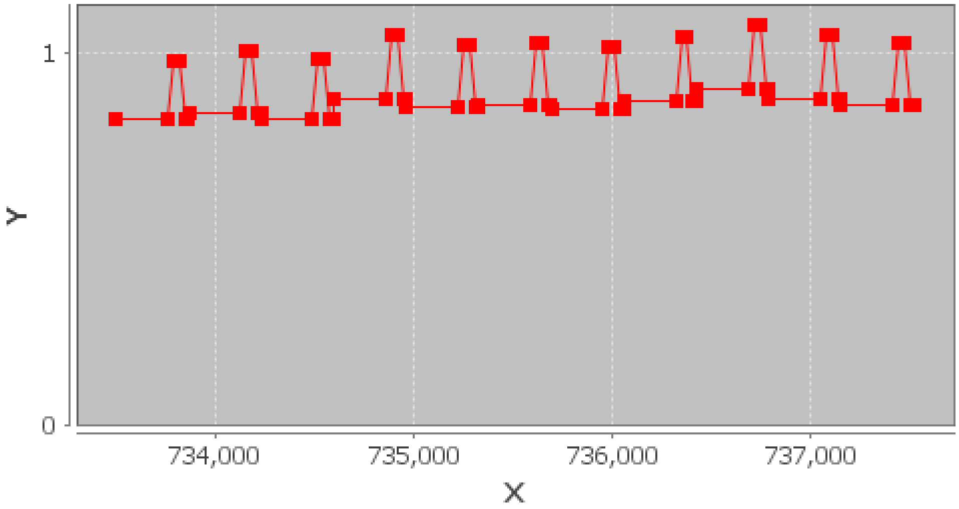<br><br>**Present In 0 Views:**<br>**Used By**<br>• Actual Public Occupancy<br>**Feedback Loops:** 0 (0.0%) (+) 0 [0,0] (-) 0 [0,0]
Core insurance model 0331	#10A, T	**Average Length of Stay _SDMlookup (Day)**<br>Average Length of Stay _SDMlookup([(733500,4)-(737517,10)],(733500,7.5),(733864,7.5),(733865,7.5),(734229,7.5),(734230,7.2),(734595,7.2),(734596,7.5),(734960,7.5),(734961,7.4),(735325,7.4),(735326,7.3),(735690,7.3),(735691,7.2),(736056,7.2),(736057,7.1),(736421,7.1),(736422,7.2),(736786,7.2),(736787,7.2),(737151,7.2),(737152,7.5),(737517,7.5))<br>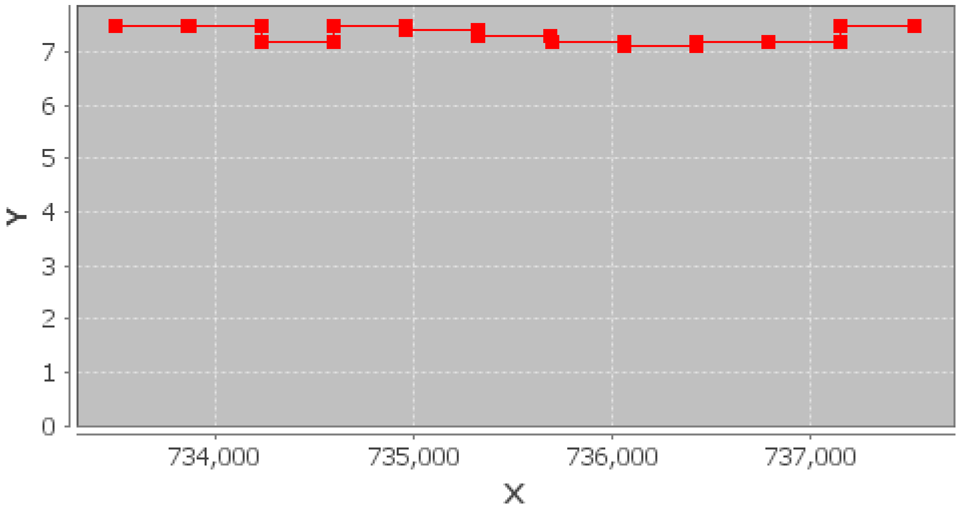<br><br>**Present In 0 Views:**<br>**Used By**<br>• Average Length of Stay<br>**Feedback Loops:** 0 (0.0%) (+) 0 [0,0] (-) 0 [0,0]

Core insurance model 0331	#15A, T	**Daily number of potentially get ill _SDMlookup** ()<br>Daily number of potentially get ill _SDMlookup([(733500,0)-(737517,10000)],(733500,3496),(733759,3496),(733789,4182),(733819,4182),(733849,3496),(733864,3496),(733865,3640),(734124,3640),(734154,4355),(734184,4355),(734214,3640),(734229,3640),(734230,3735),(734489,3735),(734519,4469),(734549,4469),(734579,3735),(734595,3735),(734596,3780),(734855,3780),(734885,4523),(734915,4523),(734945,3780),(734960,3780),(734961,3710),(735220,3710),(735250,4438),(735280,4438),(735310,3710),(735325,3710),(735326,3819),(735585,3819),(735615,4569),(735645,4569),(735675,3819),(735690,3819),(735691,3867),(735950,3867),(735980,4627),(736010,4627),(736040,3867),(736056,3867),(736057,4044),(736316,4044),(736346,4838),(736376,4838),(736406,4044),(736421,4044),(736422,4090),(736681,4090),(736711,4893),(736741,4893),(736771,4090),(736786,4090),(736787,4074),(737046,4074),(737076,4874),(737106,4874),(737136,4074),(737151,4074),(737152,3945),(737411,3945),(737441,4720),(737471,4720),(737501,3945),(737517,3945))<br>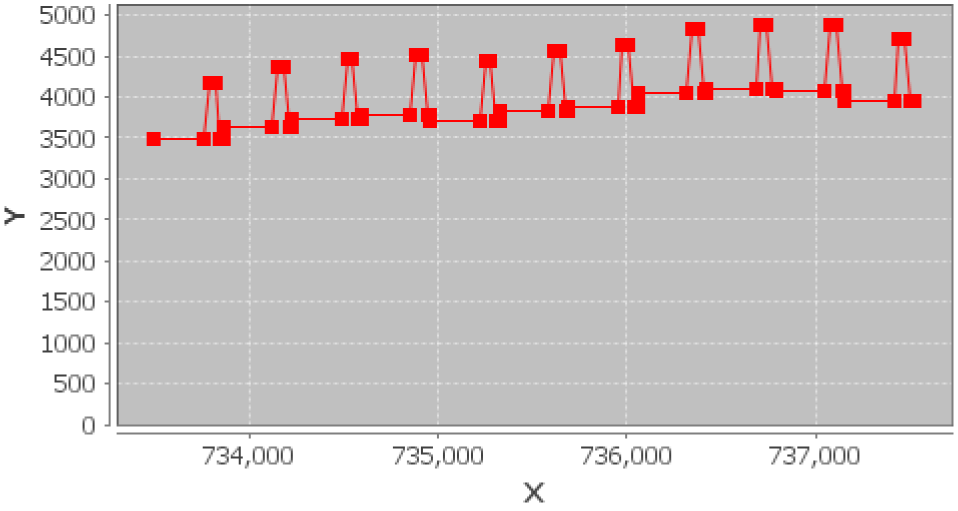<br><br>**Present In 0 Views:**<br>**Used By**<br>• Daily number of potentially get ill<br>**Feedback Loops:** 0 (0.0%) (+) 0 [0,0] (-) 0 [0,0]
Core insurance model 0331	#31A, T	**Private hospital beds _SDMlookup** **(pt)**<br>Private hospital beds _SDMlookup([(733500,0)-(737517,10000)],(733500,4022),(733774,4022),(733775,3946),(734139,3946),(734140,4098),(734504,4098),(734505,4033),(734870,4033),(734871,3882),(735235,3882),(735236,3906),(735600,3906),(735601,4014),(735965,4014),(735966,4226),(736331,4226),(736332,4644),(736696,4644),(736697,4657),(737061,4657),(737062,5056),(737426,5056),(737427,5050),(737517,5050))

		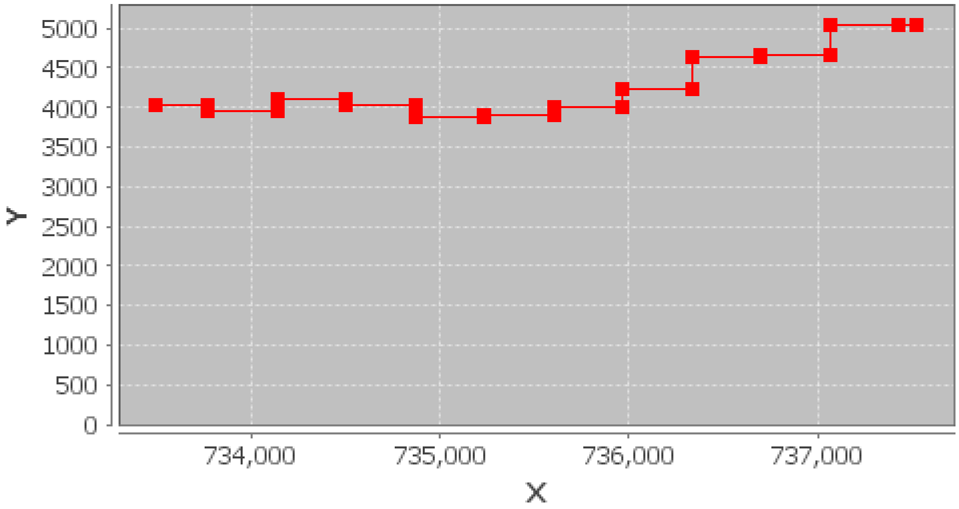<br>**Present In 0 Views:**<br>**Used By**<br>• Private hospital beds<br>**Feedback Loops:** 0 (0.0%) (+) 0 [0,0] (-) 0 [0,0]
Core insurance model 0331	#34A,T	**Public hospital beds _SDMlookup (pt)**<br>Public hospital beds _SDMlookup([(733500,0)-(737517,30000)],(733500,23046),(733864,23046),(733865,23263),(734229,23263),(734230,23286),(734595,23286),(734596,23378),(734960,23378),(734961,23686),(735325,23686),(735326,23891),(735690,23891),(735691,24161),(736056,24161),(736057,24392),(736421,24392),(736422,24621),(736786,24621),(736787,25155),(737151,25155),(737152,25661),(737517,25661))<br>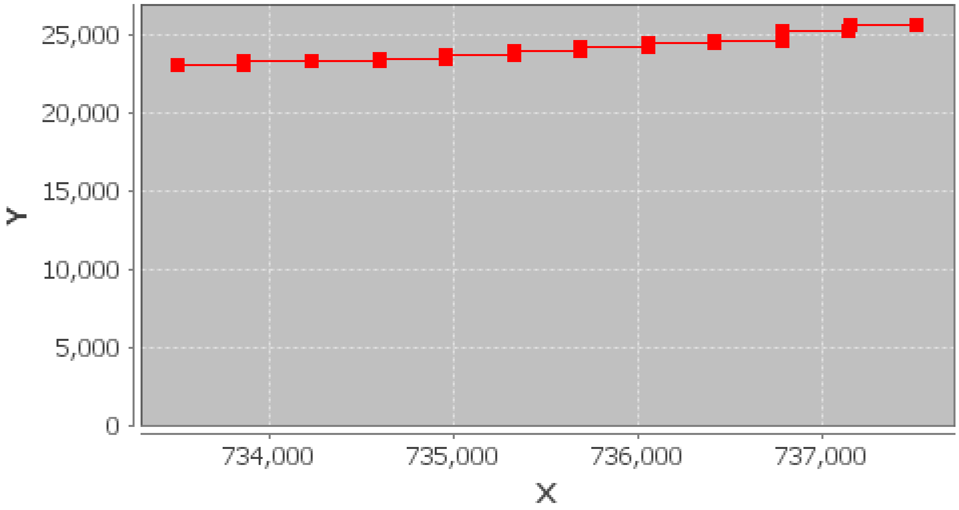<br>**Description:** 1 bed = 1 pt<br>**Present In 0 Views:**<br>**Used By**

		• Public hospital beds 1 bed = 1 pt<br><br>**Feedback Loops:** 0 (0.0%) (+) 0 [0,0] (-) 0 [0,0]
Core insurance model 0331	#39A, T	**Tax Incentive _SDMlookup ()**<br>Tax Incentive _SDMlookup([(733500,0)-(737517,10000)],(733500,0),(737151,0),(737152,8000),(737517,8000))<br>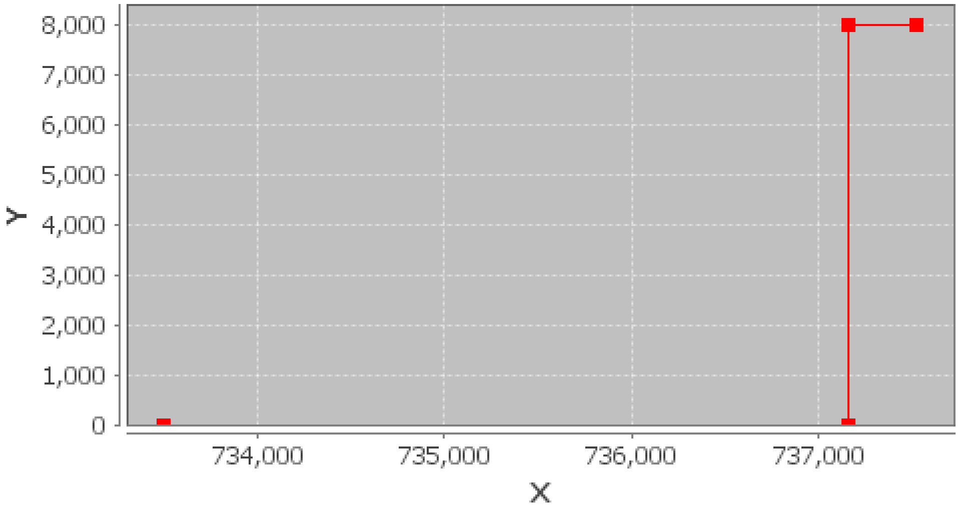<br>**Present In 0 Views:**<br><br>**Used By**<br><br>• Tax Incentive<br><br>**Feedback Loops:** 0 (0.0%) (+) 0 [0,0] (-) 0 [0,0]

Top

Quick Links:	A	B	C	D	E	F	G	H	I	J	K	L	M	N	O	P	Q	R	S	T	U	V	W	X	Y	Z

All Variables (30 Variables + 4 Control Variables)

Group	Type	**Variable**
Core insurance model 0331	A	Actual percentage of HI ()
Core insurance model 0331	A	Actual Private Hospital Use (pt)
Core insurance model 0331	A	Actual Private Occupancy ()

Core insurance model 0331	A	Actual Public Hospital Use (pt)
Core insurance model 0331	A	Actual Public Occupancy ()
Core insurance model 0331	A	Average Length of Stay (Day)
Core insurance model 0331	C	Average Premium Ratio ()
Core insurance model 0331	C	Baseline HI ()
Core insurance model 0331	L	Daily getting ill (pt)
Core insurance model 0331	F,A	Daily number of potentially get ill ()
Core insurance model 0331	C	Delay in Perception and Purchase (Day)
Core insurance model 0331	A	Diff in Occupancy ()
Core insurance model 0331	F,A	Discharged or dead from Private Hospitals (pt/Day)
Core insurance model 0331	F,A	Discharged or dead from Public Hospitals (pt/Day)
Core insurance model 0331	F,A	Getting sick and use private hospital ()
Core insurance model 0331	F,A	Getting sick and use public hospital ()
Core insurance model 0331	L	In Private Hospitals (pt)
Core insurance model 0331	L	In Public Hospitals (pt)
Core insurance model 0331	C	Occupancy to Demand ()
Core insurance model 0331	DE,A	Percentage of HI ()
Core insurance model 0331	A	Pr Occupancy ()
Core insurance model 0331	A	Private hospital beds (pt)
Core insurance model 0331	A	Pu Occupancy ()
Core insurance model 0331	A	Public hospital beds (pt)
Core insurance model 0331	C	Self pay ()
Core insurance model 0331	C	SeverityFtr ()
Core insurance model 0331	A	Tax Incentive ()
Core insurance model 0331	C	Tax Saving to demand ()
Core insurance model 0331	A	Tax Savings ()
Core insurance model 0331	C	Use HI ()
.Control	C	FINAL TIME (Day)
.Control	C	INITIAL TIME (Day)
.Control	A	SAVEPER (Day)

.Control	C	TIME STEP (Day)

Top

Variable Link Detail (30 Variables + 4 Control Variables)

Group	Type	Variable	In/Out Counts	In/Out Ratio	In Links By Polarity	Out Links By Polarity
Core insurance model 0331	InOutLinks	Percentage of HI ()	6 | 2	3.00	5| 1| 0	0| 2| 0
Core insurance model 0331	InOutLinks	Getting sick and use public hospital ()	4 | 2	2.00	1| 3| 0	1| 1| 0
Core insurance model 0331	InOutLinks	Getting sick and use private hospital ()	4 | 2	2.00	3| 1| 0	1| 1| 0
Core insurance model 0331	InOutLinks	Daily getting ill (pt)	3 | 2	1.50	1| 2| 0	2| 0| 0
Core insurance model 0331	InOutLinks	In Public Hospitals (pt)	2 | 2	1.00	1| 1| 0	2| 0| 0
Core insurance model 0331	InOutLinks	In Private Hospitals (pt)	2 | 2	1.00	1| 1| 0	2| 0| 0
Core insurance model 0331	InOutLinks	Discharged or dead from Private Hospitals (pt/Day)	3 | 1	3.00	1| 2| 0	0| 1| 0
Core insurance model 0331	InOutLinks	Average Length of Stay (Day)	2 | 2	1.00	0| 0| 2	0| 2| 0
Core insurance model 0331	InOutLinks	Tax Savings ()	2 | 1	2.00	2| 0| 0	1| 0| 0
Core insurance model 0331	InOutLinks	Tax Incentive ()	2 | 1	2.00	2| 0| 0	1| 0| 0
Core insurance model 0331	InOutLinks	Public hospital beds (pt)	2 | 1	2.00	2| 0| 0	0| 1| 0
Core insurance model 0331	InOutLinks	Pu Occupancy ()	2 | 1	2.00	1| 1| 0	1| 0| 0
Core insurance model 0331	InOutLinks	Private hospital beds (pt)	2 | 1	2.00	0| 0| 2	0| 1| 0
Core insurance model 0331	InOutLinks	Pr Occupancy ()	2 | 1	2.00	1| 1| 0	0| 1| 0

Core insurance model 0331	InOutLinks	Discharged or dead from Public Hospitals (pt/Day)	2 | 1	2.00	1| 1| 0	0| 1| 0
Core insurance model 0331	InOutLinks	Diff in Occupancy ()	2 | 1	2.00	1| 1| 0	1| 0| 0
Core insurance model 0331	InOutLinks	Daily number of potentially get ill ()	2 | 1	2.00	0| 0| 2	1| 0| 0
Core insurance model 0331	InOutLinks	Use HI ()	0 | 2	0.00	0| 0| 0	1| 1| 0
Core insurance model 0331	InOutLinks	Self pay ()	0 | 2	0.00	0| 0| 0	1| 1| 0
Core insurance model 0331	InOutLinks	Actual Public Occupancy ()	2 | 0	Infinite	0| 0| 2	0| 0| 0
Core insurance model 0331	InOutLinks	Actual Public Hospital Use (pt)	2 | 0	Infinite	0| 0| 2	0| 0| 0
Core insurance model 0331	InOutLinks	Actual Private Occupancy ()	2 | 0	Infinite	0| 0| 2	0| 0| 0
Core insurance model 0331	InOutLinks	Actual Private Hospital Use (pt)	2 | 0	Infinite	0| 0| 2	0| 0| 0
Core insurance model 0331	InOutLinks	Actual percentage of HI ()	2 | 0	Infinite	2| 0| 0	0| 0| 0
Core insurance model 0331	InOutLinks	Tax Saving to demand ()	0 | 1	0.00	0| 0| 0	1| 0| 0
Core insurance model 0331	InOutLinks	SeverityFtr ()	0 | 1	0.00	0| 0| 0	0| 1| 0
Core insurance model 0331	InOutLinks	Occupancy to Demand ()	0 | 1	0.00	0| 0| 0	0| 1| 0
Core insurance model 0331	InOutLinks	Delay in Perception and Purchase (Day)	0 | 1	0.00	0| 0| 0	1| 0| 0
Core insurance model 0331	InOutLinks	Baseline HI ()	0 | 1	0.00	0| 0| 0	1| 0| 0
Core insurance model 0331	InOutLinks	Average Premium Ratio ()	0 | 1	0.00	0| 0| 0	1| 0| 0
.Control	InOutLinks	TIME STEP (Day)	0 | 1	0.00	0| 0| 0	1| 0| 0
.Control	InOutLinks	SAVEPER (Day)	1 | 0	Infinite	1| 0| 0	0| 0| 0
.Control	InOutLinks	INITIAL TIME (Day)	(0| 0)	Infinite	0| 0| 0	0| 0| 0
.Control	InOutLinks	FINAL TIME (Day)	(0| 0)	Infinite	0| 0| 0	0| 0| 0

Top

Quick Links:	A	B	C	D	E	F	G	H	I	J	K	L	M	N	O	P	Q	R	S	T	U	V	W	X	Y	Z

Undocumented Variables (37 Variables + 0 Control Variables)

Group	Type	Variable
Core insurance model 0331	A	Actual percentage of HI ()
Core insurance model 0331	A,T	Actual percentage of HI _SDMlookup ()
Core insurance model 0331	A	Actual Private Hospital Use (pt)
Core insurance model 0331	A,T	Actual Private Hospital Use _SDMlookup (pt)
Core insurance model 0331	A	Actual Private Occupancy ()
Core insurance model 0331	A,T	Actual Private Occupancy _SDMlookup ()
Core insurance model 0331	A	Actual Public Hospital Use (pt)
Core insurance model 0331	A,T	Actual Public Hospital Use _SDMlookup (pt)
Core insurance model 0331	A	Actual Public Occupancy ()
Core insurance model 0331	A,T	Actual Public Occupancy _SDMlookup ()
Core insurance model 0331	A	Average Length of Stay (Day)
Core insurance model 0331	A,T	Average Length of Stay _SDMlookup (Day)
Core insurance model 0331	C	Average Premium Ratio ()
Core insurance model 0331	C	Baseline HI ()
Core insurance model 0331	L	Daily getting ill (pt)
Core insurance model 0331	F,A	Daily number of potentially get ill ()
Core insurance model 0331	A,T	Daily number of potentially get ill _SDMlookup ()
Core insurance model 0331	C	Delay in Perception and Purchase (Day)
Core insurance model 0331	F,A	Discharged or dead from Private Hospitals (pt/Day)
Core insurance model 0331	F,A	Discharged or dead from Public Hospitals (pt/Day)
Core insurance model 0331	F,A	Getting sick and use private hospital ()

Core insurance model 0331	F,A	Getting sick and use public hospital ()
Core insurance model 0331	L	In Private Hospitals (pt)
Core insurance model 0331	L	In Public Hospitals (pt)
Core insurance model 0331	C	Occupancy to Demand ()
Core insurance model 0331	DE,A	Percentage of HI ()
Core insurance model 0331	A	Pr Occupancy ()
Core insurance model 0331	A	Private hospital beds (pt)
Core insurance model 0331	A,T	Private hospital beds _SDMlookup (pt)
Core insurance model 0331	A	Pu Occupancy ()
Core insurance model 0331	C	Self pay ()
Core insurance model 0331	C	SeverityFtr ()
Core insurance model 0331	A	Tax Incentive ()
Core insurance model 0331	A,T	Tax Incentive _SDMlookup ()
Core insurance model 0331	C	Tax Saving to demand ()
Core insurance model 0331	A	Tax Savings ()
Core insurance model 0331	C	Use HI ()

Top

Supplementary Variables (0 Variables + 0 Control Variables)

Group	Type	**Variable**

Top

Supplementary Variables Being Used (0 Variables + 0 Control Variables)

Group	Type	**Variable**

Top

Quick Links:	A	B	C	D	E	F	G	H	I	J	K	L	M	N	O	P	Q	R	S	T	U	V	W	X	Y	Z

Unused Variables (5 Variables + 0 Control Variables)

Group	Type	Variable
Core insurance model 0331	A	Actual percentage of HI ()
Core insurance model 0331	A	Actual Private Hospital Use (pt)
Core insurance model 0331	A	Actual Private Occupancy ()
Core insurance model 0331	A	Actual Public Hospital Use (pt)
Core insurance model 0331	A	Actual Public Occupancy ()

Top

Quick Links:	A	B	C	D	E	F	G	H	I	J	K	L	M	N	O	P	Q	R	S	T	U	V	W	X	Y	Z

Stock Variables (3 Variables + 0 Control Variables)

Group	Type	Variable
Core insurance model 0331	L	Daily getting ill (pt)
Core insurance model 0331	L	In Private Hospitals (pt)
Core insurance model 0331	L	In Public Hospitals (pt)

Top

Quick Links:	A	B	C	D	E	F	G	H	I	J	K	L	M	N	O	P	Q	R	S	T	U	V	W	X	Y	Z

Equations With Embedded Data (5 Variables + 0 Control Variables)

Group	Type	Variable
Core insurance model 0331	L	Daily getting ill (pt)
Core insurance model 0331	L	In Private Hospitals (pt)
Core insurance model 0331	L	In Public Hospitals (pt)
Core insurance model 0331	DE,A	Percentage of HI ()
Core insurance model 0331	A	Tax Savings ()

Top

Quick Links:	A	B	C	D	E	F	G	H	I	J	K	L	M	N	O	P	Q	R	S	T	U	V	W	X	Y	Z

Nonmonotonic Lookup Functions (7 Variables + 0 Control Variables)

Group	Type	Variable
Core insurance model 0331	A,T	Actual Private Hospital Use _SDMlookup (pt)
Core insurance model 0331	A,T	Actual Private Occupancy _SDMlookup ()
Core insurance model 0331	A,T	Actual Public Hospital Use _SDMlookup (pt)

Core insurance model 0331	A,T	Actual Public Occupancy _SDMlookup ()
Core insurance model 0331	A,T	Average Length of Stay _SDMlookup (Day)
Core insurance model 0331	A,T	Daily number of potentially get ill _SDMlookup ()
Core insurance model 0331	A,T	Private hospital beds _SDMlookup (pt)

Top

Non-Zero End Sloped Lookup Functions (1 Variables + 0 Control Variables)

Group	Type	**Variable**	**Non-Zero**
Core insurance model 0331	Non-Zero	Actual percentage of HI _SDMlookup ()	Both

Top

Cascading Lookup Functions (0 Variables + 0 Control Variables)

Group	Type	**Variable**

Top

Equations With Step Pulse Or Related Functions (0 Variables + 0 Control Variables)

Group	Type	**Variable**

Top

Equations With If Then Else Functions (0 Variables + 0 Control Variables)

Group	Type	Variable

Top

Equations With Min Or Max Functions (0 Variables + 0 Control Variables)

Group	Type	Variable

Top

Complex Variable (Richardson's Rule Threshold = 3) (3 Variables + 0 Control Variables)

Group	Type	Variable	Complexity
Core insurance model 0331	Complexity	Getting sick and use private hospital ()	4
Core insurance model 0331	Complexity	Getting sick and use public hospital ()	4
Core insurance model 0331	Complexity	Percentage of HI ()	6

Top

Complex Stock (0 Variables + 0 Control Variables)

Group	Type	Variable

Top

**Quick Links:**	A	B	C	D	E	F	G	H	I	J	K	L	M	N	O	P	Q	R	S	T	U	V	W	X	Y	Z

State Variables (4 Variables + 0 Control Variables)

Group	Type	**Variable**
Core insurance model 0331	L	Daily getting ill (pt)
Core insurance model 0331	L	In Private Hospitals (pt)
Core insurance model 0331	L	In Public Hospitals (pt)
Core insurance model 0331	DE,A	Percentage of HI ()

Top

Variables With Source Information (0 Variables + 0 Control Variables)

Group	Type	**Variable**	**Sources**

Top

Variables With Dimensionless Units (0 Variables + 0 Control Variables)

Group	Type	**Variable**

Top

Quick Links:	A	B	C	D	E	F	G	H	I	J	K	L	M	N	O	P	Q	R	S	T	U	V	W	X	Y	Z

Variables without Predefined Min or Max Values (30 Variables + 4 Control Variables)

Group	Type	Variable
Core insurance model 0331	A	Actual percentage of HI ()
Core insurance model 0331	A	Actual Private Hospital Use (pt)
Core insurance model 0331	A	Actual Private Occupancy ()
Core insurance model 0331	A	Actual Public Hospital Use (pt)
Core insurance model 0331	A	Actual Public Occupancy ()
Core insurance model 0331	A	Average Length of Stay (Day)
Core insurance model 0331	C	Average Premium Ratio ()
Core insurance model 0331	C	Baseline HI ()
Core insurance model 0331	L	Daily getting ill (pt)
Core insurance model 0331	F,A	Daily number of potentially get ill ()
Core insurance model 0331	C	Delay in Perception and Purchase (Day)
Core insurance model 0331	A	Diff in Occupancy ()
Core insurance model 0331	F,A	Discharged or dead from Private Hospitals (pt/Day)
Core insurance model 0331	F,A	Discharged or dead from Public Hospitals (pt/Day)
Core insurance model 0331	F,A	Getting sick and use private hospital ()
Core insurance model 0331	F,A	Getting sick and use public hospital ()
Core insurance model 0331	L	In Private Hospitals (pt)
Core insurance model 0331	L	In Public Hospitals (pt)
Core insurance model 0331	C	Occupancy to Demand ()
Core insurance model 0331	DE,A	Percentage of HI ()
Core insurance model 0331	A	Pr Occupancy ()
Core insurance model 0331	A	Private hospital beds (pt)
Core insurance model 0331	A	Pu Occupancy ()

Core insurance model 0331	A	Public hospital beds (pt)
Core insurance model 0331	C	Self pay ()
Core insurance model 0331	C	SeverityFtr ()
Core insurance model 0331	A	Tax Incentive ()
Core insurance model 0331	C	Tax Saving to demand ()
Core insurance model 0331	A	Tax Savings ()
Core insurance model 0331	C	Use HI ()
.Control	C	FINAL TIME (Day)
.Control	C	INITIAL TIME (Day)
.Control	A	SAVEPER (Day)
.Control	C	TIME STEP (Day)

Top

Function Sensitivity Parameters (0 Variables + 0 Control Variables)

Group	Type	**Variable**

Top

Data Lookup Tables (0 Variables + 0 Control Variables)

Group	Type	**Variable**

Top

Variables Using Macros (0 Variables + 0 Control Variables)

Group	Type	**Variable**

Top

**Quick Links:**	A	B	C	D	E	F	G	H	I	J	K	L	M	N	O	P	Q	R	S	T	U	V	W	X	Y	Z

Variables Not In Any View (0 Variables + 4 Control Variables)

Group	Type	**Variable**
.Control	C	FINAL TIME (Day)
.Control	C	INITIAL TIME (Day)
.Control	A	SAVEPER (Day)
.Control	C	TIME STEP (Day)

Top

Equations With Unit Errors Or Warnings (26 Variables + 0 Control Variables)

Group	Type	**Variable**	**Units**
Core insurance model 0331	Units	Actual percentage of HI ()	Lhs Units: None Specified
Core insurance model 0331	Units	Actual Private Hospital Use (pt)	Lhs Units: (pt)<br>Rhs Units: LOOKUP Used With Dimensioned Argument < (Day) ><br>Complete Rhs Units: LOOKUP (pt , Day)
Core insurance model 0331	Units	Actual Private Occupancy ()	Lhs Units: None Specified

Core insurance model 0331	Units	Actual Public Hospital Use (pt)	Lhs Units: (pt)<br>Rhs Units: LOOKUP Used With Dimensioned Argument < (Day) ><br>Complete Rhs Units: LOOKUP (pt , Day)
Core insurance model 0331	Units	Actual Public Occupancy ()	Lhs Units: None Specified
Core insurance model 0331	Units	Average Length of Stay (Day)	Lhs Units: (Day)<br>Rhs Units: LOOKUP Used With Dimensioned Argument < (Day) ><br>Complete Rhs Units: LOOKUP (Day , Day)
Core insurance model 0331	Units	Average Premium Ratio ()	Lhs Units: None Specified
Core insurance model 0331	Units	Baseline HI ()	Lhs Units: None Specified
Core insurance model 0331	Units	Daily number of potentially get ill ()	Lhs Units: None Specified
Core insurance model 0331	Units	Diff in Occupancy ()	Lhs Units: None Specified
Core insurance model 0331	Units	Getting sick and use private hospital ()	Lhs Units: None Specified
Core insurance model 0331	Units	Getting sick and use public hospital ()	Lhs Units: None Specified
Core insurance model 0331	Units	In Private Hospitals (pt)	Lhs Units: (pt)<br>Rhs Units: INTEG ((Error >>> (Dmnl) - (pt/Day) <<< Error) , (constant))<br>Complete Rhs Units: INTEG ((Dmnl - pt/Day) , 0.0)
Core insurance model 0331	Units	In Public Hospitals (pt)	Lhs Units: (pt)<br>Rhs Units: INTEG ((Error >>> (Dmnl) - (pt/Day) <<< Error) , (constant))<br>Complete Rhs Units: INTEG ((Dmnl - pt/Day) , 0.0)
Core insurance model 0331	Units	Occupancy to Demand ()	Lhs Units: None Specified
Core insurance model 0331	Units	Percentage of HI ()	Lhs Units: None Specified
Core insurance model 0331	Units	Pr Occupancy ()	Lhs Units: None Specified
Core insurance model 0331	Units	Private hospital beds (pt)	Lhs Units: (pt)<br>Rhs Units: LOOKUP Used With Dimensioned

			Argument < (Day) ><br>Complete Rhs Units: LOOKUP (pt , Day)
Core insurance model 0331	Units	Pu Occupancy ()	Lhs Units: None Specified
Core insurance model 0331	Units	Public hospital beds (pt)	Lhs Units: (pt)<br>Rhs Units: LOOKUP Used With Dimensioned Argument < (Day) ><br>Complete Rhs Units: LOOKUP (pt , Day)
Core insurance model 0331	Units	Self pay ()	Lhs Units: None Specified
Core insurance model 0331	Units	SeverityFtr ()	Lhs Units: None Specified
Core insurance model 0331	Units	Tax Incentive ()	Lhs Units: None Specified
Core insurance model 0331	Units	Tax Saving to demand ()	Lhs Units: None Specified
Core insurance model 0331	Units	Tax Savings ()	Lhs Units: None Specified
Core insurance model 0331	Units	Use HI ()	Lhs Units: None Specified

Top

Units (2/1)

Units	Type	Alternates
Day	Basic	
pt	Basic	
pt/Day	Combined	

Top

Feedback Loops (6|0 Loops) Maximum Loop Length: 5 [2,5] | [0,0]

Group	Type	Variable	Loops	+	-	+/- Ratio	?	Loops (IVV)	+	-	+/- Ratio	?
Core insurance model 0331	Feedback..	Daily getting ill (pt)	2 (33.3%)	0 [0, 0]	2 [2, 2]	0.00	0 [0, 0]	0 (0 %)	0 [0, 0]	0 [0, 0]	NA	0 [0, 0]
Core insurance model 0331	Feedback..	Diff in Occupancy ()	2 (33.3%)	1 [5, 5]	1 [5, 5]	1.00	0 [0, 0]	0 (0 %)	0 [0, 0]	0 [0, 0]	NA	0 [0, 0]
Core insurance model 0331	Feedback..	Getting sick and use private hospital ()	2 (33.3%)	1 [5, 5]	1 [2, 2]	1.00	0 [0, 0]	0 (0 %)	0 [0, 0]	0 [0, 0]	NA	0 [0, 0]
Core insurance model 0331	Feedback..	Getting sick and use public hospital ()	2 (33.3%)	0 [0, 0]	2 [2, 5]	0.00	0 [0, 0]	0 (0 %)	0 [0, 0]	0 [0, 0]	NA	0 [0, 0]
Core insurance model	Feedback..	In Private Hospitals (pt)	2 (33.3%)	1 [5, 5]	1 [2, 2]	1.00	0 [0, 0]	0 (0 %)	0 [0, 0]	0 [0, 0]	NA	0 [0, 0]

0331												
Core insurance model 0331	Feedback..	In Public Hospitals (pt)	2 (33.3%)	0 [0, 0]	2 [2, 5]	0.00	**0** [0, 0]	0 (0%)	0 [0, 0]	0 [0, 0]	NA	**0** [0, 0]
Core insurance model 0331	Feedback..	Percentage of HI ()	2 (33.3%)	1 [5, 5]	1 [5, 5]	1.00	**0** [0, 0]	0 (0%)	0 [0, 0]	0 [0, 0]	NA	**0** [0, 0]
Core insurance model 0331	Feedback..	Discharged or dead from Private Hospitals (pt/Day)	1 (16.7%)	0 [0, 0]	1 [2, 2]	0.00	**0** [0, 0]	0 (0%)	0 [0, 0]	0 [0, 0]	NA	**0** [0, 0]
Core insurance model 0331	Feedback..	Discharged or dead from Public Hospitals (pt/Day)	1 (16.7%)	0 [0, 0]	1 [2, 2]	0.00	**0** [0, 0]	0 (0%)	0 [0, 0]	0 [0, 0]	NA	**0** [0, 0]
Core insurance model 0331	Feedback..	Pr Occupancy ()	1 (16.7%)	1 [5, 5]	0 [0, 0]	Infinite	**0** [0, 0]	0 (0%)	0 [0, 0]	0 [0, 0]	NA	**0** [0, 0]

Core insurance model 0331	Feedback..	Pu Occupancy ()	1 (16.7%)	0 [0, 0]	1 [5, 5]	0.00	0 [0, 0]	0 (0%)	0 [0, 0]	0 [0, 0]	NA	0 [0, 0]
Core insurance model 0331	Feedback..	Actual percentage of HI ()	0 (0%)	0 [0, 0]	0 [0, 0]	NA	0 [0, 0]	0 (0%)	0 [0, 0]	0 [0, 0]	NA	0 [0, 0]
Core insurance model 0331	Feedback..	Actual Private Hospital Use (pt)	0 (0%)	0 [0, 0]	0 [0, 0]	NA	0 [0, 0]	0 (0%)	0 [0, 0]	0 [0, 0]	NA	0 [0, 0]
Core insurance model 0331	Feedback..	Actual Private Occupancy ()	0 (0%)	0 [0, 0]	0 [0, 0]	NA	0 [0, 0]	0 (0%)	0 [0, 0]	0 [0, 0]	NA	0 [0, 0]
Core insurance model 0331	Feedback..	Actual Public Hospital Use (pt)	0 (0%)	0 [0, 0]	0 [0, 0]	NA	0 [0, 0]	0 (0%)	0 [0, 0]	0 [0, 0]	NA	0 [0, 0]

Core insurance model 0331	Feedback..	Actual Public Occupancy ()	0 (0%)	0 [0, 0]	0 [0, 0]	NA	0 [0, 0]	0 (0%)	0 [0, 0]	0 [0, 0]	NA	0 [0, 0]
Core insurance model 0331	Feedback..	Average Length of Stay (Day)	0 (0%)	0 [0, 0]	0 [0, 0]	NA	0 [0, 0]	0 (0%)	0 [0, 0]	0 [0, 0]	NA	0 [0, 0]
Core insurance model 0331	Feedback..	Average Premium Ratio ()	0 (0%)	0 [0, 0]	0 [0, 0]	NA	0 [0, 0]	0 (0%)	0 [0, 0]	0 [0, 0]	NA	0 [0, 0]
Core insurance model 0331	Feedback..	Baseline HI ()	0 (0%)	0 [0, 0]	0 [0, 0]	NA	0 [0, 0]	0 (0%)	0 [0, 0]	0 [0, 0]	NA	0 [0, 0]
Core insurance model 0331	Feedback..	Daily number of potentially get ill ()	0 (0%)	0 [0, 0]	0 [0, 0]	NA	0 [0, 0]	0 (0%)	0 [0, 0]	0 [0, 0]	NA	0 [0, 0]

Core insurance model 0331	Feedback..	Delay in Perception and Purchase (Day)	0 (0%)	0 [0, 0]	0 [0, 0]	NA	**0** [0, 0]	0 (0%)	0 [0, 0]	0 [0, 0]	NA	**0** [0, 0]
Core insurance model 0331	Feedback..	Occupancy to Demand ()	0 (0%)	0 [0, 0]	0 [0, 0]	NA	**0** [0, 0]	0 (0%)	0 [0, 0]	0 [0, 0]	NA	**0** [0, 0]
Core insurance model 0331	Feedback..	Private hospital beds (pt)	0 (0%)	0 [0, 0]	0 [0, 0]	NA	**0** [0, 0]	0 (0%)	0 [0, 0]	0 [0, 0]	NA	**0** [0, 0]
Core insurance model 0331	Feedback..	Public hospital beds (pt)	0 (0%)	0 [0, 0]	0 [0, 0]	NA	**0** [0, 0]	0 (0%)	0 [0, 0]	0 [0, 0]	NA	**0** [0, 0]
Core insurance model 0331	Feedback..	Self pay ()	0 (0%)	0 [0, 0]	0 [0, 0]	NA	**0** [0, 0]	0 (0%)	0 [0, 0]	0 [0, 0]	NA	**0** [0, 0]

Core insurance model 0331	Feedback..	Severity Ftr ()	0 (0%)	0 [0, 0]	0 [0, 0]	NA	**0** [0, 0]	0 (0%)	0 [0, 0]	0 [0, 0]	NA	**0** [0, 0]
Core insurance model 0331	Feedback..	Tax Incentive ()	0 (0%)	0 [0, 0]	0 [0, 0]	NA	**0** [0, 0]	0 (0%)	0 [0, 0]	0 [0, 0]	NA	**0** [0, 0]
Core insurance model 0331	Feedback..	Tax Saving to demand ()	0 (0%)	0 [0, 0]	0 [0, 0]	NA	**0** [0, 0]	0 (0%)	0 [0, 0]	0 [0, 0]	NA	**0** [0, 0]
Core insurance model 0331	Feedback..	Tax Savings ()	0 (0%)	0 [0, 0]	0 [0, 0]	NA	**0** [0, 0]	0 (0%)	0 [0, 0]	0 [0, 0]	NA	**0** [0, 0]
Core insurance model 0331	Feedback..	Use HI ()	0 (0%)	0 [0, 0]	0 [0, 0]	NA	**0** [0, 0]	0 (0%)	0 [0, 0]	0 [0, 0]	NA	**0** [0, 0]

.Control	Feedback..	FINAL TIME (Day)	0 (0%)	0 [0, 0]	0 [0, 0]	NA	0 [0, 0]	0 (0%)	0 [0, 0]	0 [0, 0]	NA	0 [0, 0]
.Control	Feedback..	INITIAL TIME (Day)	0 (0%)	0 [0, 0]	0 [0, 0]	NA	0 [0, 0]	0 (0%)	0 [0, 0]	0 [0, 0]	NA	0 [0, 0]
.Control	Feedback..	SAVEPER (Day)	0 (0%)	0 [0, 0]	0 [0, 0]	NA	0 [0, 0]	0 (0%)	0 [0, 0]	0 [0, 0]	NA	0 [0, 0]
.Control	Feedback..	TIME STEP (Day)	0 (0%)	0 [0, 0]	0 [0, 0]	NA	0 [0, 0]	0 (0%)	0 [0, 0]	0 [0, 0]	NA	0 [0, 0]

Top

Exogenous Variables Analysis (19 Variables + 4 Control Variables)

Group	Type	**Variable**	**Variable Number**	**Data Source**
Core insurance model 0331	Exogenous	Actual percentage of HI ()	1	1 Exogenous
Core insurance model 0331	Exogenous	Actual Private Hospital Use (pt)	2	1 Exogenous
Core insurance model 0331	Exogenous	Actual Private Occupancy ()	3	1 Exogenous
Core insurance model 0331	Exogenous	Actual Public Hospital Use (pt)	4	1 Exogenous
Core insurance model 0331	Exogenous	Actual Public Occupancy ()	5	1 Exogenous
Core insurance model 0331	Exogenous	Average Length of Stay (Day)	6	1 Exogenous
Core insurance model 0331	Exogenous	Average Premium Ratio ()	7	Hardcoded
Core insurance model 0331	Exogenous	Baseline HI ()	8	Hardcoded
Core insurance model 0331	Exogenous	Daily number of potentially get ill ()	9	1 Exogenous

Core insurance model 0331	Exogenous	Delay in Perception and Purchase (Day)	10	Hardcoded
Core insurance model 0331	Exogenous	Occupancy to Demand ()	11	Hardcoded
Core insurance model 0331	Exogenous	Private hospital beds (pt)	12	1 Exogenous
Core insurance model 0331	Exogenous	Public hospital beds (pt)	13	1 Exogenous
Core insurance model 0331	Exogenous	Self pay ()	14	Hardcoded
Core insurance model 0331	Exogenous	SeverityFtr ()	15	Hardcoded
Core insurance model 0331	Exogenous	Tax Incentive ()	16	1 Exogenous
Core insurance model 0331	Exogenous	Tax Saving to demand ()	17	Hardcoded
Core insurance model 0331	Exogenous	Tax Savings ()	18	2 Exogenous
Core insurance model 0331	Exogenous	Use HI ()	19	Hardcoded
.Control	Exogenous	FINAL TIME (Day)	20	Hardcoded
.Control	Exogenous	INITIAL TIME (Day)	21	Hardcoded
.Control	Exogenous	SAVEPER (Day)	22	1 Exogenous
.Control	Exogenous	TIME STEP (Day)	23	Hardcoded

Top

Endogenous Variables Analysis (11 Variables + 0 Control Variables) (Maximum Endogenous Path Length: 5)

Group	Type	**Variable**	**Variable Number**	**Input Links**	**Direct Exogenous**	**Percent Exogenous**	**Indirect Exogenous**	**Indirect Minimum**	**Indirect Mean**	**Indirect Median**	**Indirect Maximum**	**Exogenous Unconnected**	**Exogenous Connected**

Core insurance model 0331	Endogenous	Percentage of HI ()	1	6	5	83.3	4	2	4.25	5.00	5	16	9
Core insurance model 0331	Endogenous	Discharged or dead from Private Hospitals (pt/Day)	2	3	2	66.7	7	3	3.86	4.00	5	16	9
Core insurance model 0331	Endogenous	Discharged or dead from Public Hospitals (pt/Day)	3	2	1	50.0	7	3	3.86	4.00	5	17	8
Core insurance model 0331	Endogenous	Getting sick and use private hospital ()	4	4	2	50.0	5	2	2.20	2.00	3	18	7
Core insurance model 0331	Endogenous	Getting sick and use public hospital ()	5	4	2	50.0	5	2	2.20	2.00	3	18	7
Core insurance model 0331	Endogenous	Pr Occupancy ()	6	2	1	50.0	8	3	3.75	4.00	5	16	9
Core insurance model 0331	Endogenous	Pu Occupancy ()	7	2	1	50.0	7	3	3.86	4.00	5	17	8
Core insurance mode	Endogenous	Daily getting ill (pt)	8	3	1	33.3	7	2	2.86	3.00	4	17	8

l 0331													
Core insurance model 0331	Endogenous	Diff in Occupancy ()	9	2	0	0.0	7	4	4.57	5.00	5	18	7
Core insurance model 0331	Endogenous	In Private Hospitals (pt)	10	2	0	0.0	8	2	2.75	3.00	4	17	8
Core insurance model 0331	Endogenous	In Public Hospitals (pt)	11	2	0	0.0	7	2	2.86	3.00	4	18	7

Top

Macros (0 Variables)

Name	Macro Definition	Expanded Macro Definition

Top

**Quick Links:**	A	B	C	D	E	F	G	H	I	J	K	L	M	N	O	P	Q	R	S	T	U	V	W	X	Y	Z

Positive Polarity Causal Links (26 Variables)

Cause	Effect	**Polarity**
Actual percentage of HI _SDMlookup	Actual percentage of HI	+
Average Premium Ratio	Tax Savings	+
Baseline HI	Percentage of HI	+
Daily getting ill	Getting sick and use private hospital	+
Daily getting ill	Getting sick and use public hospital	+
Daily number of potentially get ill	Daily getting ill	+
Delay in Perception and Purchase	Percentage of HI	+
Diff in Occupancy	Percentage of HI	+
Getting sick and use private hospital	In Private Hospitals	+
Getting sick and use public hospital	In Public Hospitals	+
In Private Hospitals	Discharged or dead from Private Hospitals	+
In Private Hospitals	Pr Occupancy	+
In Public Hospitals	Discharged or dead from Public Hospitals	+
In Public Hospitals	Pu Occupancy	+
Pu Occupancy	Diff in Occupancy	+
Public hospital beds _SDMlookup	Public hospital beds	+
Self pay	Getting sick and use private hospital	+
Tax Incentive	Tax Savings	+
Tax Incentive _SDMlookup	Tax Incentive	+
Tax Saving to demand	Percentage of HI	+
Tax Savings	Percentage of HI	+
Time	Actual percentage of HI	+
Time	Public hospital beds	+
Time	Tax Incentive	+
TIME STEP	SAVEPER	+
Use HI	Getting sick and use private hospital	+

Top

Quick Links:	A	B	C	D	E	F	G	H	I	J	K	L	M	N	O	P	Q	R	S	T	U	V	W	X	Y	Z

Negative Polarity Causal Links (15 Variables)

Cause	Effect	Polarity
Average Length of Stay	Discharged or dead from Private Hospitals	-
Average Length of Stay	Discharged or dead from Public Hospitals	-
Discharged or dead from Private Hospitals	In Private Hospitals	-
Discharged or dead from Public Hospitals	In Public Hospitals	-
Getting sick and use private hospital	Daily getting ill	-
Getting sick and use public hospital	Daily getting ill	-
Occupancy to Demand	Percentage of HI	-
Percentage of HI	Getting sick and use private hospital	-
Percentage of HI	Getting sick and use public hospital	-
Pr Occupancy	Diff in Occupancy	-
Private hospital beds	Pr Occupancy	-
Public hospital beds	Pu Occupancy	-
Self pay	Getting sick and use public hospital	-
SeverityFtr	Discharged or dead from Private Hospitals	-
Use HI	Getting sick and use public hospital	-

Top

Quick Links:	A	B	C	D	E	F	G	H	I	J	K	L	M	N	O	P	Q	R	S	T	U	V	W	X	Y	Z

Function-based Polarity Causal Links (14 Variables)

Cause	Effect	**Polarity**
Actual Private Hospital Use _SDMlookup	Actual Private Hospital Use	Lookup Inconsistent
Actual Private Occupancy _SDMlookup	Actual Private Occupancy	Lookup Inconsistent
Actual Public Hospital Use _SDMlookup	Actual Public Hospital Use	Lookup Inconsistent
Actual Public Occupancy _SDMlookup	Actual Public Occupancy	Lookup Inconsistent
Average Length of Stay _SDMlookup	Average Length of Stay	Lookup Inconsistent
Daily number of potentially get ill _SDMlookup	Daily number of potentially get ill	Lookup Inconsistent
Private hospital beds _SDMlookup	Private hospital beds	Lookup Inconsistent
Time	Actual Private Hospital Use	Lookup Inconsistent
Time	Actual Private Occupancy	Lookup Inconsistent
Time	Actual Public Hospital Use	Lookup Inconsistent
Time	Actual Public Occupancy	Lookup Inconsistent
Time	Average Length of Stay	Lookup Inconsistent
Time	Daily number of potentially get ill	Lookup Inconsistent
Time	Private hospital beds	Lookup Inconsistent

Top

Rate-to-rate Links (0 Variables)

Cause	Effect

Top

View-Variable Profile

View	View-Variable Profile
Not in View	4Variables (8.7%)
View 1	30Variables (65.2%)

Top

List Of 2 views and their 34 Variables

	Not in View	View 1	
**Total:**	**4**	**30**	**Total:**
SeverityFtr (In 1 View)		■	SeverityFtr (In 1 View)
Discharged or dead from Private Hospitals (In 1 View)		■	Discharged or dead from Private Hospitals (In 1 View)
Getting sick and use private hospital (In 1 View)		■	Getting sick and use private hospital (In 1 View)
Getting sick and use public hospital (In 1 View)		■	Getting sick and use public hospital (In 1 View)
Pu Occupancy (In 1 View)		■	Pu Occupancy (In 1 View)
Pr Occupancy (In 1 View)		■	Pr Occupancy (In 1 View)
In Private Hospitals (In 1 View)		■	In Private Hospitals (In 1 View)
In Public Hospitals (In 1 View)		■	In Public Hospitals (In 1 View)
Discharged or dead from Public Hospitals (In 1 View)		■	Discharged or dead from Public Hospitals (In 1 View)
Daily number of potentially get ill (In 1 View)		■	Daily number of potentially get ill (In 1 View)
Percentage of HI (In 1 View)		■	Percentage of HI (In 1 View)
Daily getting ill (In 1 View)		■	Daily getting ill (In 1 View)
Actual percentage of HI (In 1 View)		■	Actual percentage of HI (In 1 View)
Tax Savings (In 1 View)		■	Tax Savings (In 1 View)

Tax Incentive (In 1 View)		■	Tax Incentive (In 1 View)
Use HI (In 1 View)		■	Use HI (In 1 View)
Tax Saving to demand (In 1 View)		■	Tax Saving to demand (In 1 View)
Average Premium Ratio (In 1 View)		■	Average Premium Ratio (In 1 View)
Occupancy to Demand (In 1 View)		■	Occupancy to Demand (In 1 View)
Actual Private Hospital Use (In 1 View)		■	Actual Private Hospital Use (In 1 View)
Actual Private Occupancy (In 1 View)		■	Actual Private Occupancy (In 1 View)
Actual Public Hospital Use (In 1 View)		■	Actual Public Hospital Use (In 1 View)
Actual Public Occupancy (In 1 View)		■	Actual Public Occupancy (In 1 View)
Baseline HI (In 1 View)		■	Baseline HI (In 1 View)
Self pay (In 1 View)		■	Self pay (In 1 View)
Average Length of Stay (In 1 View)		■	Average Length of Stay (In 1 View)
Delay in Perception and Purchase (In 1 View)		■	Delay in Perception and Purchase (In 1 View)
Diff in Occupancy (In 1 View)		■	Diff in Occupancy (In 1 View)
Private hospital beds (In 1 View)		■	Private hospital beds (In 1 View)
Public hospital beds (In 1 View)		■	Public hospital beds (In 1 View)
FINAL TIME (In 1 View)	■		FINAL TIME (In 1 View)
INITIAL TIME (In 1 View)	■		INITIAL TIME (In 1 View)
SAVEPER (In 1 View)	■		SAVEPER (In 1 View)
TIME STEP (In 1 View)	■		TIME STEP (In 1 View)
**Total:**	**4**	**30**	**Total:**
	Not in View	View 1	

Source File: C:\Users\chenj297\Downloads\Core insurance model 0331.mdl (Sat Apr 02 16:45:29 CST 2022)
Report Created On Sat Apr 02 16:46:02 CST 2022
SDM-Doc Tool Version 1.4.7
Global Security Sciences Division
Argonne National Laboratory
