# Supplementary material for: The Feedback Loop Between the Demand for Voluntary Private Insurance and the Burden of Healthcare System: An Explanatory System Dynamics Model of Hong Kong
Source: Int J Health Policy Manag. 2022 Jul 6;11(12):3040–51. doi: 10.34172/ijhpm.2022.6738 (PMC10105189; doi:10.34172/ijhpm.2022.6738)

**Article title:** The Feedback Loop Between the Demand for Voluntary Private Insurance and the Burden of Healthcare System: An Explanatory System Dynamics Model of Hong Kong

**Journal name:** International Journal of Health Policy and Management (IJHPM)

**Authors' information**: Yiran Zou[1], Junqiao Chen[2,3,4]*

[1]Center for Data Science, New York University, New York City, NY, USA

[2]Roche Hong Kong and Macau, Hong Kong SAR, China

[3]University Institute of Lisbon (ISCTE-IUL), Lisbon, Portugal

[4]Faculty of Sciences, University of Lisbon, Lisbon, Portugal

(*Corresponding author: junqiao.chen@roche.com)

**Supplementary file 3.** Graphical Results During Calibration

**Figure S1:** Graphical result for simulated percentage of health insurance vs. reality when calibrating for (a) adding MAX to formula (7); (b) OccuDmd; (c) SeverityFtr.

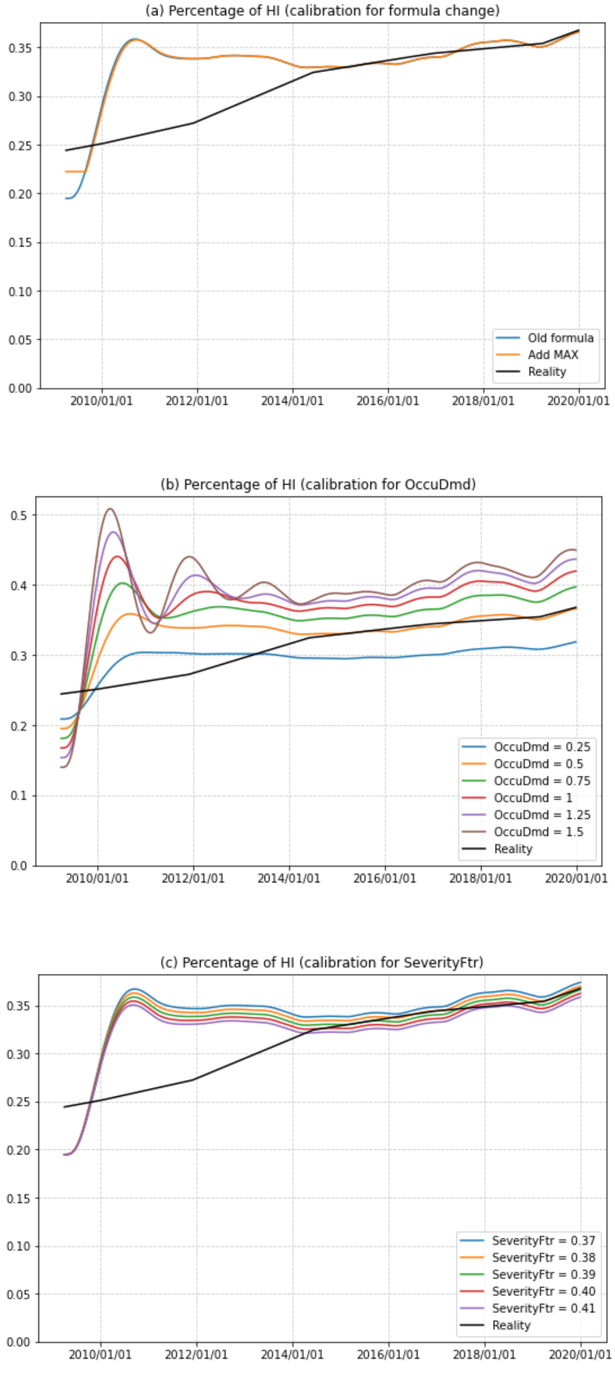

Supplement: Supplementary file 3 — Graphical Results During Calibration. [file ijhpm-11-3040-s003.pdf]
